# The Development and Validation of the Pornography Use in Romantic Relationships Scale

**DOI:** 10.1007/s10508-023-02534-5

**Published:** 2023-02-28

**Authors:** Nicholas J. Lawless, Gery C. Karantzas, Laura Knox

**Affiliations:** School of Psychology, Deakin University, 221 Burwood Hwy, Burwood, Melbourne, VIC 3125 Australia

**Keywords:** Romantic relationships, Factor analysis, Pornography Use in Romantic Relationships Scale

## Abstract

Recent reviews of the pornography literature have called for the development of valid and reliable measures that assess multiple facets of pornography use. Moreover, despite pornography use having important implications for romantic relationships, there are currently no self-report assessments of pornography use specifically within the context of romantic relationships. To address these limitations, the current paper reports on two studies regarding the development and psychometric evaluation of a 38-item multidimensional measure of pornography use within the context of romantic relationships: the Pornography Use in Romantic Relationships Scale (PURRS). Study 1 (*n* = 739) reports on an Exploratory and Confirmatory Factor Analytic approach to determine the factor structure of the PURRS. Study 2 (*n* = 765) reports on the cross-validation of the factor structure of the PURRS, before assessing the criterion validity of the measure. The PURRS exhibited good internal consistency, construct validity, and criterion validity. The findings suggest that the PURRS is best modeled by 13 first-order factors, though a higher-order factor structure comprising four broad factors may also be used. The PURRS significantly extends on past assessments of pornography use, and in particular, advances the assessment and study of pornography use within the context of romantic relationships.

**Supplementary Information:**

The online version contains supplementary material available at 10.1007/s10508-023-02534-5.

## Introduction

Over the past decade, systematic reviews (Kohut et al., 2019; Marshall & Miller, 2019; Short et al., 2012) have highlighted major problems regarding the assessment of pornography use. First, measures do not provide respondents with a definition of pornography use, so it is unclear what respondents are referring to when answering questions, and thus, whether the results from studies using one measure can be compared to studies that use another measure. Second, there is a conspicuous lack of reliable and valid measures of pornography use. Of the measures that have been developed, most are derived to meet specific study aims, and details regarding the psychometrics of these measures are scant. Third, despite the multidimensional nature of pornography use, existing measures typically assess only one aspect of pornography use (usually frequency). Therefore, the reviews conclude that there is a need to develop and psychometrically evaluate a multidimensional measure of pornography use that also provides a definition of pornography use to respondents. In addition, pornography use in romantic relationships is commonplace, with roughly 70% of men and 34% of women in romantic relationships using pornography every year (Willoughby & Busby, 2016). However, no scale has been developed to measure pornography use specifically within the context of romantic relationships. To address these gaps, this paper reports on the development and evaluation of the Pornography Use in Romantic Relationships Scale (PURRS). We begin by explicating these measurement problems, before describing the development and evaluation of the PURRS across two studies.

### The Need to Provide Respondents with a Definition of Pornography

The first prominent problem in the literature is that most studies measuring pornography use do not provide a definition of pornography use to respondents (Kohut et al., 2019; Marshall & Miller, 2019; Short et al., 2012). Because of this, there is no way of knowing what respondents consider as constituting pornography use when rating survey items; thus, there are inherent inconsistencies across the measurement of pornography in the literature. Moreover, in the rare cases when a definition of pornography is provided to respondents, there are considerable differences between studies as to how pornography is defined (Kohut et al., 2019; Marshall & Miller, 2019; Short et al., 2012). Some definitions of pornography include nudity whilst some do not; some include the sexually arousing function of pornography, yet other definitions omit this function; some restrict which mediums are considered pornography (such as excluding written text), whilst others do not; some definitions consider interactive sexual experiences (such as live online chats or in-person lap dances) as pornography and other definitions do not. This variation in the definition of pornography reflects a broad lack of consensus in the field regarding the definition of pornography (Kohut et al., 2019; Willoughby & Busby, 2016). To resolve this problem and to be able to effectively compare results across studies, the field must reach a consensus regarding the definition of pornography and ensure that this definition is presented to respondents as part of pornography use measures (Kohut et al., 2019; Marshall & Miller, 2019; Short et al., 2012).

Following a detailed analysis of the definition of pornography as part of their review of the literature, Kohut et al. (2019) proposed the following definition:

Using pornography means to intentionally look at, read, or listen to: (a) pictures, videos, or films that depict nude individuals or people having sex; or (b) written or audio material that describes nude individuals, or people having sex. Using pornography does not involve viewing or interacting with actual, live, nude individuals, or participating in interactive sexual experiences with other human beings in person or online. For example, participating in live sex chat or a camshow, and getting a “lapdance” in a strip club are not considered pornography use (p. 16).

This definition is an important step forward in developing a definition to present to respondents. Firstly, according to this definition, media does not need to depict sexual behavior to be considered pornography. Instead, it need only depict nudity, something which aligns with the views of many laypeople (Kohut, 2014; Kohut et al., 2019; Willoughby & Busby, 2016). Secondly, this definition encompasses all mediums of pornography (e.g., written or audio). Constructing a definition of pornography which incorporates multiple modalities is wise in order to capture the full range of consumers, particularly women who in many cases only use written pornography (Solano et al., 2020). Thirdly, this definition not only defines pornography, but also what it means to use pornography. The term use implies intentionality on behalf of the user, more so than other terms such as consumption or exposure. Use is also able to encompass the various mediums that may constitute pornography (e.g., written or audio) which terms like view and watch cannot accommodate. Finally, this definition clearly excludes live, interactive experiences, given that these are considerably different in nature to typical conceptualizations of pornography (Kohut et al., 2019). However, despite its many strengths, there are two limitations with this definition.

First, this definition does not make explicit the sexually arousing nature of pornographic material. Nevertheless, sexual arousal is widely acknowledged as the primary function of pornography use (Emmers-Sommer, 2018; Grubbs et al., 2019; Solano et al., 2020). Not including the sexually arousing function of pornography in a definition means that medical textbooks (with images of naked bodies) and classic artworks depicting nudity (e.g., Michelangelo’s Statue of David or The Last Judgment) would be considered pornographic. Other reviews of the literature have highlighted the importance of including the function of pornography when providing a definition (Marshall & Miller, 2019; Short et al., 2012). Moreover, a panel of leading pornography experts from a range of disciplines were asked about the definition of pornography, and the majority endorsed a definition that included the sexually arousing function of pornography (McKee et al., 2020). In line with this, prominent researchers across both early and more recent studies in the field of pornography have included the sexually arousing function of pornography in their various working definitions of the construct (Ashton et al., 2019; Hald et al., 2010; Lambert et al., 2012; Malamuth, 1993; Wright et al., 2017; Zillmann & Bryant, 1988). Finally, research investigating lay people’s concept of pornography found that most lay people understand pornography to be material which is likely to trigger sexual arousal by depicting sexual behavior and nudity (Kohut, 2014; Kohut et al., 2019). Thus, including the sexually arousing function of pornography is necessary, as it not only provides an important conceptual distinction, but it also brings the definition of pornography use into alignment with layperson and expert accounts of what constitutes pornography.

Secondly, the phrase “people having sex” is problematic in that it is quite restrictive in terms of what is considered pornographic. For example, within the context of this phrasing, depictions of a man or woman masturbating alone would not be considered pornographic. However, research suggests that a large proportion of people consider depictions of solo masturbation as pornographic (e.g., Willoughby & Busby, 2016). A way forward to address this limitation is to substitute the phrase “people having sex” with “explicit sexual behavior,” a term which clearly encompasses sexual activity aside from sexual intercourse and has often been used in past definitions of pornography (e.g., Wright et al., 2017).

In light of the definitional issues noted above, we propose the following definition:

Using pornography means to intentionally look at, watch, read, or listen to sexually arousing material (pictures, videos, films, written text or audio) which depicts nudity and/or explicit sexual behavior. This does not include participating in interactive sexual experiences in person or online, such as a “lap-dance” in a strip club or a live sex chat.

This more succinct definition retains all the strengths of the guiding definition provided by Kohut et al. (2019; see above), whilst also including the sexually arousing function of pornography and encompassing sexual activity other than sex, such as masturbation. In doing so, this definition embodies lay peoples’ concept of pornography as well as that of leading pornography researchers.

### The Need for Reliable, Valid Measures Which Assess Multiple Facets of Pornography Use

Given the lack of consensus regarding the definition of pornography to date, it is unsurprising that the measurement of this construct also varies considerably between studies. Systematic reviews of the literature have found that the vast majority of studies use researcher-generated questions to measure pornography use (Kohut et al., 2019; Marshall & Miller, 2019; Short et al., 2012). These measures are typically single-item self-report instruments that vary in response options (e.g., dichotomous (yes/no) response scales, Likert scales, frequency scales), the time-span of pornography use (e.g., past month, past 6 months, ever used), and the medium of pornographic material consumed (e.g., written text, X-rated videos, internet pornography). Moreover, the few multi-item measures which have undergone psychometric evaluation typically focus on only one aspect of pornography use. For instance, measures have now been developed which assess pornography addiction (Bőthe et al., 2018; Kor et al., 2014), motives to use pornography (Bőthe et al., 2020) and self-perceived effects of pornography use (Miller et al., 2019a, 2019b). Whilst these types of measures have utility in research and clinical settings that are specifically interested in these particular aspects of pornography use, this research also highlights that the phenomenon of pornography use is multidimensional. To this end, it is important that the conceptualization and measurement of the construct take a multidimensional perspective.

In response to the lack of multidimensional assessment, two new measures—the Pornography Usage Measure (PUM; Busby et al., 2020) and the Consumption of Pornography Scale-General (COPS-G; Hatch et al., 2020)—have been developed that do take a multidimensional approach to the assessment of pornography, and in doing so, warrant further discussion. Although these newly developed measures improve on past measures of pornography use, they ultimately fall short of addressing a number of the limitations in the conceptualization and measurement of pornography use noted as part of recent reviews of the field (Kohut et al., 2019; Marshall & Miller, 2019; Short et al., 2012).

Both the PUM and the COPS-G assess the frequency of pornography use, provide evidence regarding the reliability and validity of the measures, and consider the pornography content being used. However, these measures fall short in a number of ways. First, both the PUM and the COPS-G draw seemingly arbitrary distinctions in the type of pornographic content assessed. The PUM distinguishes between content primarily by level of explicitness, and the COPS-G (apart from four items asking about S&M pornography) distinguishes between content by asking about the gender and number of people depicted in the pornography being viewed. In both cases, the authors provide no justification for making these distinctions. As Kohut et al. (2019) outline in their review, distinctions between content should be based on theoretical reasoning, not merely observable differences.

Second, neither the PUM nor the COPS-G assesses the context in which pornography is used, such as whether pornography is used for masturbation or joint use with a romantic partner. Instead, the PUM has a large focus on how “sexualized” the pornography is (referred to as “selectivity”; Busby et al., 2020) and the COPS-G places much emphasis on accidental exposure to pornography; neither of which have been a major focus of the literature to date. Furthermore, the scale authors do not provide compelling reasons to assess these aspects of pornography use.

Finally, the PUM and the COPS-G do not provide any definition of pornography to respondents. Thus, despite improving on past measures by assessing multiple facets of pornography use (including an attempt to consider the content of the pornography used), the PUM and the COPS-G ultimately fall short of the recommendations outlined in recent reviews of the literature (Kohut et al., 2019; Marshall & Miller, 2019; Short et al., 2012). Specifically, the development of a valid and reliable measure that accounts for several important aspects of pornography use and provides respondents with a definition of pornography use.

### The Current Study

In brief, recent reviews (Kohut et al., 2019; Marshall & Miller, 2019; Short et al., 2012) agree that there is a need for psychometrically sound measures to be developed that comprehensively assess the multifaceted nature of pornography use whilst providing respondents with a definition of the construct. However, recent measures have not addressed this entire suite of issues. In addition, no scale has been developed to measure pornography use specifically within the context of romantic relationships. Nevertheless, understanding the impact of pornography use within this context has been identified as an area in need of additional research (Willoughby et al., 2020). Even if pornography use is typically a solo endeavor, those in romantic relationships are likely to use pornography for different reasons (e.g., in place of sex with one’s partner), in different contexts (e.g., joint use with one’s partner), and with different consequences (e.g., conflict with one’s partner) than those who are single. In brief, pornography use is more than simply an individual behavior occurring within a relationship; rather, it is “best seen as a relationship phenomenon” (p. 580, Brown et al., 2017). Thus, in order to effectively study this relationship phenomenon, there is a need for a scale which measures pornography use specifically in the context of romantic relationships.

Consequently, the aim of the current paper was to develop and psychometrically evaluate a multidimensional measure of pornography use within the context of romantic relationships, titled the Pornography Use in Romantic Relationships Scale (PURRS). To address this aim, the current paper reports on two studies. Study 1 reports on the development of the item pool for the measure and the construct validity of the PURRS. Study 2 reports on a cross-validation of the structure of the measure and extends the psychometric evaluation of the PURRS by assessing criterion-related validity.

### Study 1

**Fig. 1:**
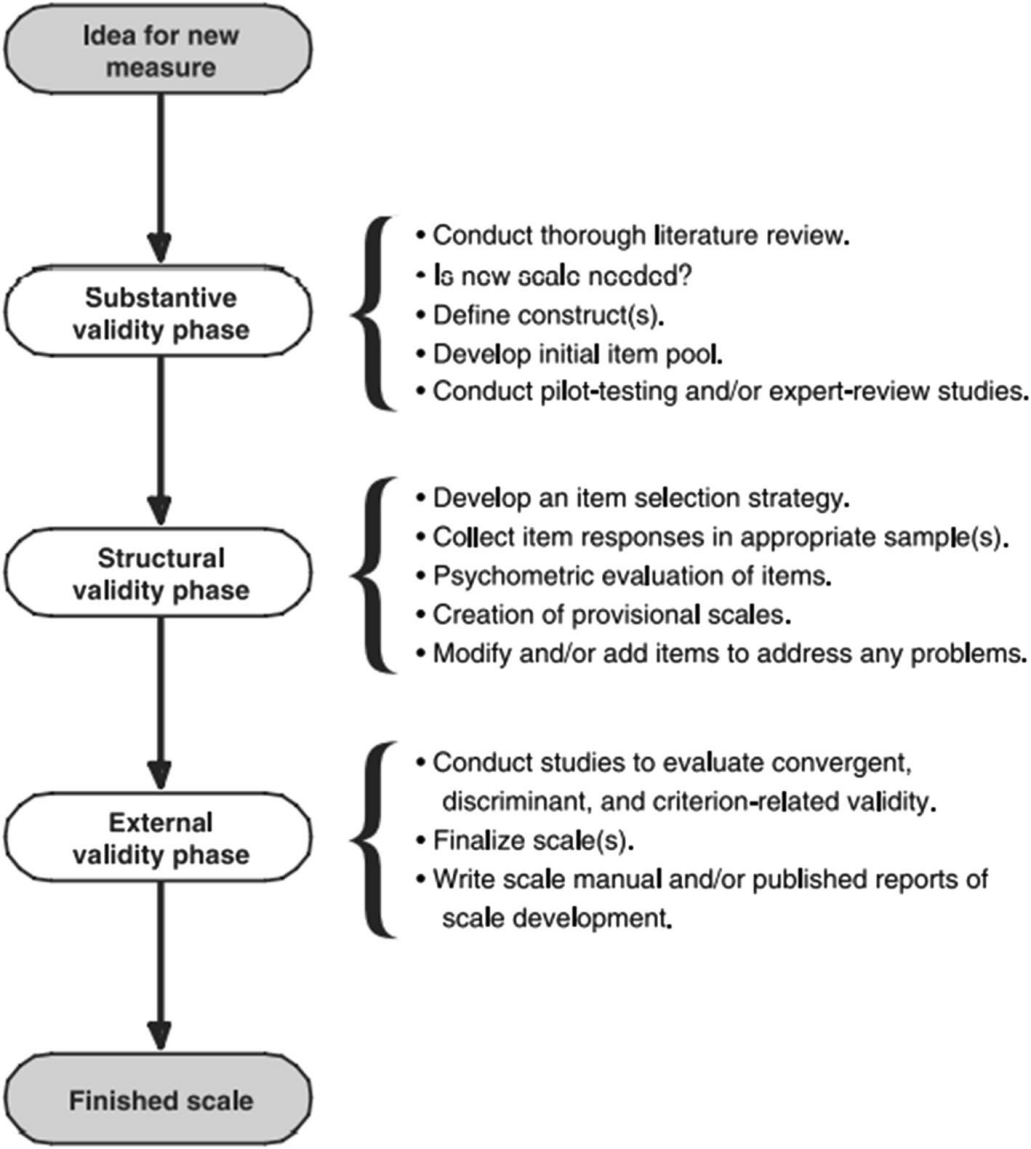
Flowchart depicting phases of scale development, as found in Simms (2008; reused with permission)

Study 1 reports on the development and construct validation of the PURRS. The measure was developed in accordance with best-practice guidelines as recommended by Simms (2008; see Fig. 1).

The first phase, dubbed the substantive validity phase, included a thorough literature review to determine the existence of established measures, their adequacy in measuring the construct, and whether there was a need for the development of a new measure (reasons that were addressed in the introduction of this paper). Based on this literature review, it was identified that there was indeed a need to develop a new measure, and the measurement development stage began with the generation of an initial item pool.

### Overview of Item Pool Development

The first step in the development of the measure was creating an item pool which reflected the multidimensional nature of pornography use in romantic relationships. Two criteria were used to guide the development of the item pool. First, items were included which were deemed relevant to pornography use specifically in the context of romantic relationships, given the intended focus of the measure. Second, each item needed to relate to an individual’s own pornography use, rather than general thoughts or evaluative judgments about pornography use in general (such as pornography acceptance) or consequences of pornography use (such as feelings of guilt or shame).

In addition to meeting these criteria, it was essential that items captured a series of dimensions which have been consistently identified as important aspects of pornography use in romantic relationships. A full list of these factors is presented in Table 1, along with example research, which has either examined the factor empirically or acknowledged the factor to be conceptually important. This list is not exhaustive, but the exemplar citations provided in Table 1 evidence the acknowledgement of these factors in recent studies of pornography use. A description of each of the factors is presented in Table 1 along with a rationale for the inclusion of the respective factor.

**Table 1:** Important factors to include in the measure based on the past research

Broad domain	Specific factor	Examples of past research highlighting this
Drive to Use	Frequency	(e.g., Kohut et al., (2019); Marshall & Miller, (2019))
	Craving	(e.g., Allen et al., 2017; Kraus & Rosenberg, 2014; Snagowski et al., 2016)
	Attractive porn	(e.g., Grov et al., 2011; Kohut et al., 2017; Séguin et al., 2018; Staley & Prause, 2013)
Unhealthy context	Secrecy	(e.g., Kohut et al., 2017; Resch & Alderson, 2014; Willoughby & Leonhardt, 2020)
	Masturbation	(e.g., Emmers-Sommer, 2018; Ley, 2019; Miller, McBain, et al., 2019; Prause, 2019)
	Prefer porn	(e.g., Berger et al., 2019; Kohut et al., 2017; Wright et al., 2019)
	Replace partner	(e.g., Kohut et al., 2017; Perry, 2019; Watson & Smith, 2012)
Perceived Positives	Joint use	(e.g., Bridges & Morokoff, 2011; Kohut et al., 2018; Maddox et al., 2011)
	Relational content	(e.g., Bridges et al., 2010; Kohut et al., 2019; Liberman, 2015; Young, 2014)
	Sex education	(e.g., Kohut et al., 2017; Peter & Valkenburg, 2006; Rothman & Adhia, 2016; Wright et al., 2019)
Harmful Content	Aggressive content	(e.g., Bridges et al., 2010; Hald et al., 2010; Wright et al., 2016)
	Nonconsensual content	(e.g., Cusack & Waranius, 2012; Klaassen & Peter, 2015; Kohut et al., 2019; Willoughby et al., 2020)
	Nonmonogamous content	(e.g., Rasmussen et al., 2019; Willoughby et al., 2020)

To organize the multitude of dimensions, factors are grouped into four overarching themes: drive to use pornography, using pornography in an unhealthy context, perceived positives of pornography use, and using pornography that depicts harmful content.

Drive to use refers to aspects which increase or signify one’s proclivity to use pornography. Reasons for measuring one’s drive to use pornography are twofold. Firstly, the more one uses pornography, the more likely it is to influence attitudes and behavior through repetitive exposure (Wright, 2011 Secondly, someone who is highly driven to use pornography is likely to harbor an appetitive motivation for their pornography use that is greater than someone who experiences a low drive to use (Sklenarik et al., 2019). The majority of pornography research to date measures drive to use in the form of frequency of pornography use (Kohut et al., 2019; Marshall & Miller, 2019). However, in addition to frequency, another factor that reflects a person’s drive to use pornography is craving (i.e., the extent to which one craves pornography use). It is acknowledged that many people experience cravings for pornography use, to the extent that a specific measure of pornography craving termed the Pornography Craving Questionnaire (Kraus & Rosenberg, 2014) was developed. Lastly, perceiving those in pornography to be sexually attractive and good at sex (what we have termed attractive porn) is likely to provoke sexual desire and, in turn, would increase one’s drive to use pornography. To the best of the authors’ knowledge, no empirical research has yet been conducted which assesses attractive porn. Yet, it is assumed that a person’s drive to use pornography is motivated by a degree of attraction to those depicted in pornography. It seems particularly pertinent to capture this aspect of pornography use when measuring pornography use in the context of romantic relationships, as an individual’s degree of attraction to those depicted in pornography may result in the development of unrealistic expectations of sexual partners (Kohut et al., 2017; Séguin et al., 2018), dissatisfaction with one’s partner (Weaver et al., 1984) and conflict within relationships (Grov et al., 2011; Regnerus, 2019). Evidently, it is important for a measure of pornography use in romantic relationships to assess frequency, craving, and attractive porn, aspects which all reflect one’s drive to use.

The extent to which pornography is likely to impact users not only depends on their drive to use, but also the extent to which one is using pornography in an unhealthy context, especially for those in romantic relationships (Campbell & Kohut, 2017; Kohut et al., 2019; Willoughby et al., 2020). Unhealthy contexts may include using pornography in secrecy, for solo masturbation, in preference to sexual experiences with a partner (i.e., prefer porn), or in place of the love and intimacy provided by a partner (i.e., replace partner). These unhealthy contexts are likely to play a large part in determining the effect of pornography use on relationship outcomes. For example, lying or hiding pornography use from one’s partner (i.e., secrecy) may result in a romantic partner feeling betrayed and distrustful, whereas openly disclosing pornography use to a romantic partner may attenuate such negative outcomes (Resch & Alderson, 2014; Willoughby & Leonhardt, 2020). A person who masturbates whilst using pornography may be less motivated to have sex with their partner if they have already experienced sexual release (Ley et al., 2014; Paterson et al., 2014; Perry, 2019). Pornography use may lead one to develop a preference for the use of pornography over sexual intimacy with one’s partner or find pornography more arousing than having sex with one’s partner (i.e., prefer porn, e.g., Berger et al., 2019; Kohut et al., 2017; Wright et al., 2019). Alternatively, some may use pornography because their partner does not want to have sex or because they feel their partner does not love them (i.e., replace partner, e.g., Kohut et al., 2017; Perry, 2019). It is assumed that both using porn in preference to or in place of a romantic partner leads to less emotional and sexual intimacy between partners and thus can be considered unhealthy contexts. Hence, secrecy, masturbation, prefer porn, and replace partner represent a number of unhealthy contexts to be assessed by a measure of pornography use in romantic relationships.

Conversely, there are some contexts in which pornography use is perceived to be positive for those in a romantic relationship (i.e., perceived positives). For instance, it has been argued that joint use—the term employed to describe using pornography with one’s romantic partner—leads to more open communication within couples (Kohut et al., 2018). Some have also argued that using pornography which depicts positive behaviors (e.g., non-sexual physical affection, having sex to express love; Liberman, 2015; Young, 2014) or for the purposes of sexual education (Hesse & Pedersen, 2017; Rissel et al., 2017) may yield benefits for romantic relationships. The only way to test these assumptions is to include these factors when assessing pornography use. Indeed, a common criticism leveled at pornography research is that it is typically harm-focused and does not allow for the possibility of finding positive effects of pornography use (e.g., Campbell & Kohut, 2017). Thus, any measurement of pornography use must assess factors which are potentially positive for romantic relationships, including joint use, relational content, and sex education.

Finally, a theme that is frequently discussed in the pornography literature is the extent to which someone uses pornography depicting harmful content. Negative relationship behaviors such as verbal and physical aggression (i.e., aggressive content), sexual coercion (i.e., nonconsensual content), and infidelity (i.e., nonmonogamous content) are known to be harmful to romantic relationships, and content analyses suggest that these behaviors are commonly depicted in mainstream pornography (Bridges et al., 2010; Cusack & Waranius, 2012; Rasmussen et al., 2019). Sexual script theory asserts that using harmful pornographic content is likely to increase one’s acceptance of these behaviors and, in turn, increase the likelihood of enacting these behaviors in their own relationship. As one review concluded: “Content, in a word, is king: it is believed to influence learning, inclination to enact, attitudes about actions, perceived norms, arousal, affect, and sexual standards and expectations” (p 13, Kohut et al., 2019). Therefore, a measure of pornography use in romantic relationships needs to measure the extent that one uses pornography depicting aggressive content, nonconsensual content, and nonmonogamous content.

Following the identification of the 13 dimensions outlined above (presented in Table 1), an aspect of the substantive validity phase was to generate a series of items to capture each of these 13 factors. In total, 51 items were generated to capture the full list of dimensions outlined in Table 1. Some of the items were adapted or based on existing measures (such as the Pornography Craving Questionnaire; Kraus & Rosenberg, 2014). However, the majority of items were developed by the authors in order to assess dimensions of pornography use that were not addressed by any previous measures. All items were rated on a 7-point scale, in line with the recommendations provided by Simms et al. (2019). This not only provided consistency and ease of scoring and subscale comparisons, but provided a response format that effectively distinguishes between varying levels of item endorsement whilst not providing too few or too many response options. The scale anchors differed depending on what was deemed as the most appropriate way of assessing a given set of items (e.g., frequency, attitudinal).

The item pool was subjected to a face validity and acceptability stage in which a pilot testing of all 51 items was conducted with experts (*n* = 2) and lay people (*n* = 7) who provided feedback in relation to item clarity, the concept the item was intended to measure, and the suitability of the pornography definition and response formats provided. This resulted in several minor adjustments, including the re-wording of items deemed confusing or increasing the clarity of instructions, and modifying some anchors to ensure they were polar opposites (e.g., a little to extremely, instead of not at all to extremely).

### Participants

A total of 739 participants (76.2% men, 21.7% women, 2.2% identified as another gender) took part in the study. To participate, people needed to be at least 18 years of age, fluent in English, and in their current relationship for at least six months. Participants also needed to have used pornography in the past year to be included in the study. Participants ranged from 18 to 72 years of age (*M* = 28.60, *SD* = 7.81) and had been in a romantic relationship for an average of just over five and a half years (*M* = 5.62, *SD* = 5.48). Sexual orientation was predominantly heterosexual (76.4%) and bisexual (17.3%). The sample comprised of people who were married (31.4%), engaged (8.8%), cohabiting (26.9%), steadily dating (30.1%) and casually dating (2.7%). Participants were mostly Caucasian (79.7%), Asian (10.2%), and Hispanic (6.6%) and were primarily living in the USA (47.6%), Australia (13.3%), Canada (9.3%), and the UK (8.4%). Approximately three-quarters of the sample reported completing further study following high school, and 80% reported that they did not follow any religion.

### Materials and Procedure

Following ethics approval from the University’s Human Research Ethics Committee, participants were recruited via social networking sites and forums on the topics of sex, pornography, psychology, and relationships (e.g., Facebook, Reddit). A link was provided, which took participants to the study’s Plain Language Statement (PLS). The PLS was a comprehensive document containing all the study details such as aims and procedures. After viewing the PLS and providing their consent, participants completed a series of demographic questions and the 51 pornography items via an anonymous online survey using the survey software Qualtrics. The definition of pornography use (outlined in the introduction) was provided to respondents prior to completing the items and, again, midway through the measure. There were no incentives offered to participants for completing the study.

### Data Analysis

**Fig. 2:**
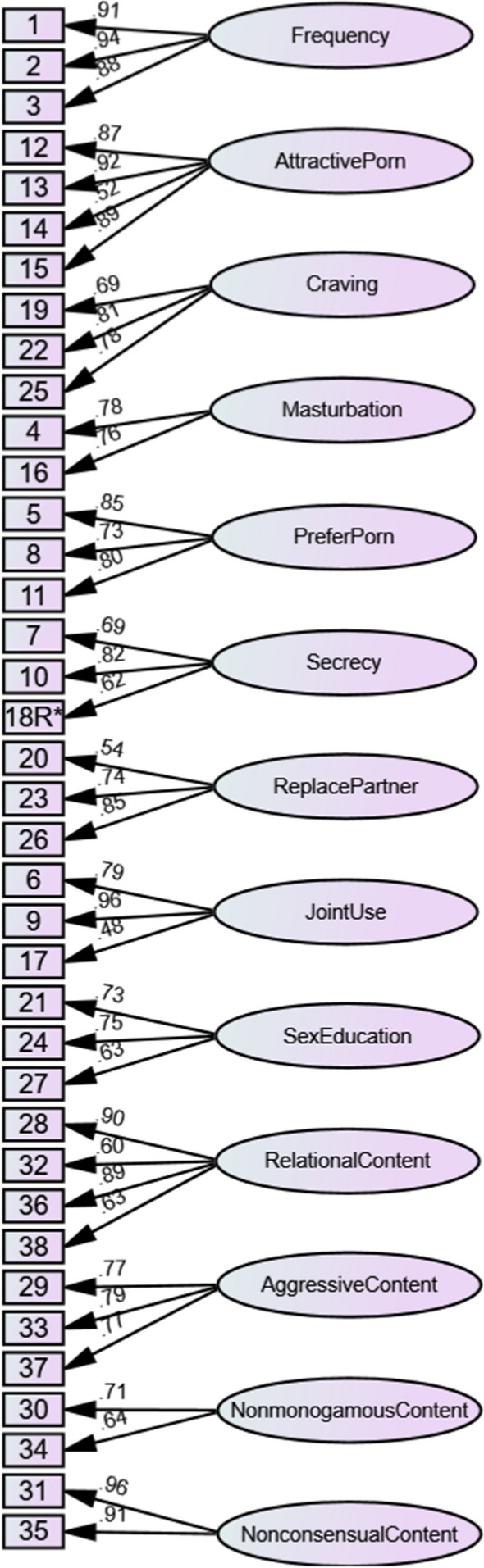
For ease of interpretation, error terms and correlations between first-order factors have been removed. The factor correlations are presented in Table 3

**Fig. 3:**
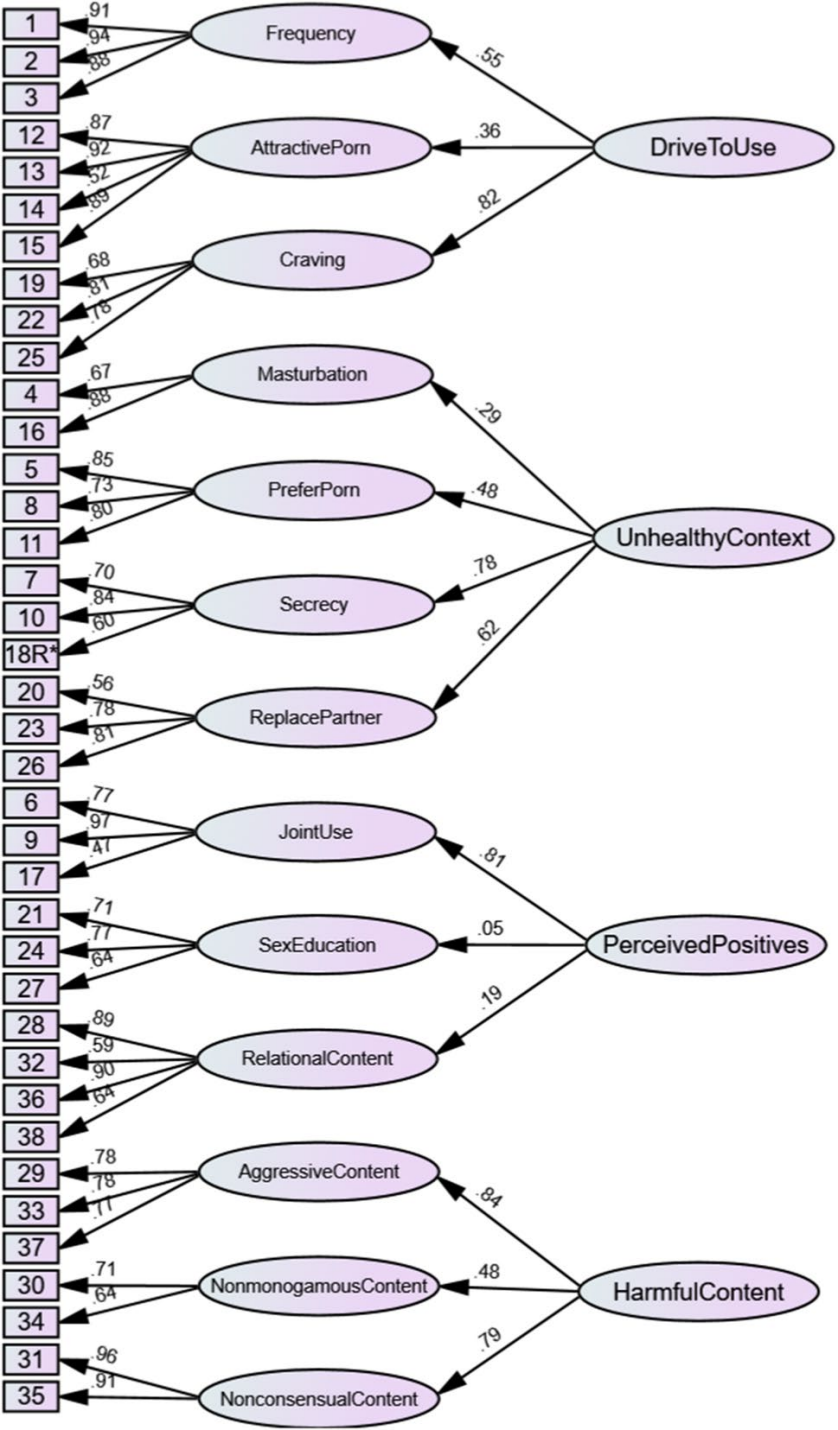
For ease of interpretation, error terms and correlations between second-order factors have been removed. The factor correlations are presented in Table 4

**Fig. 4:**
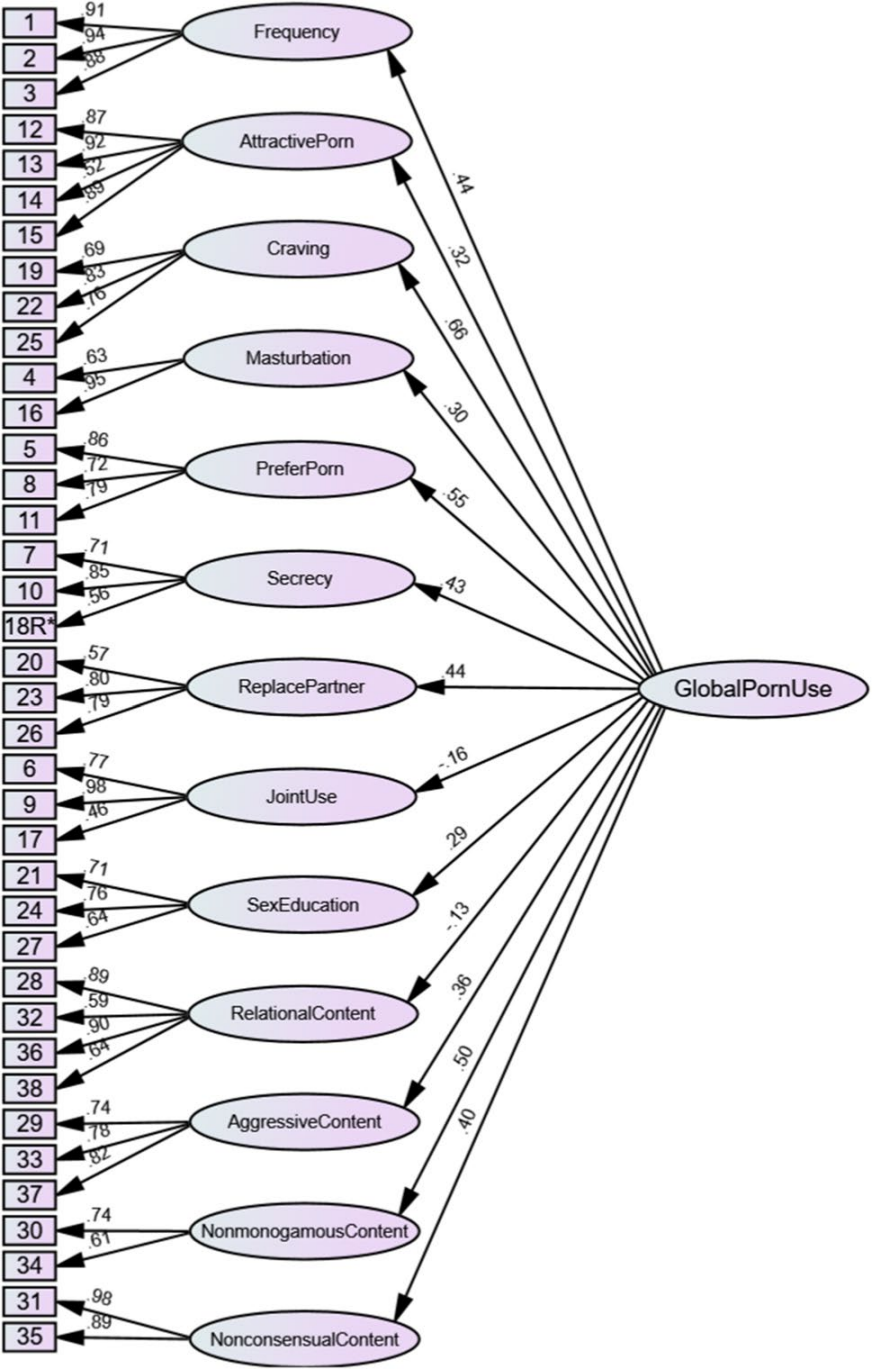
For ease of interpretation, error terms have been removed

**Fig. 5:**
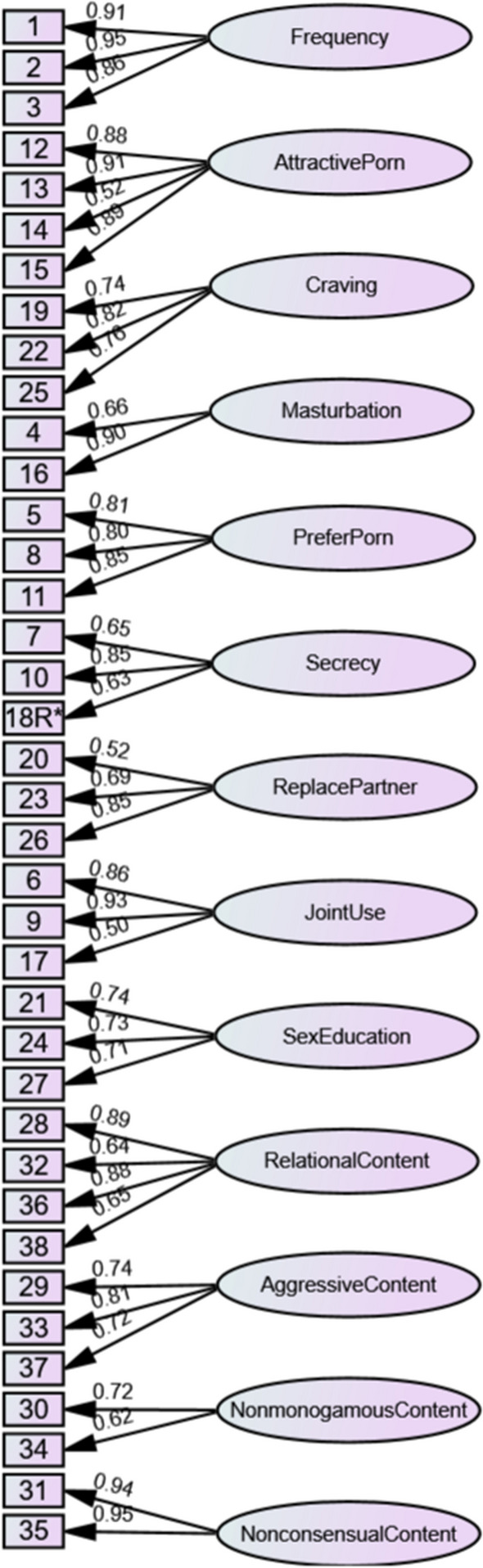
For ease of interpretation, error terms and correlations between first-order factors have been removed

Upon completion of the data collection, the data were first subjected to exploratory factor analysis (EFA) using maximum likelihood estimation with oblique rotation as an initial step in delineating the factor structure of the measure. Confirmatory factor analysis (CFA) with maximum likelihood estimation (Muthén & Kaplan, 1985) was then used as a subsequent step in the psychometric evaluation process to further assess the construct validity of the scale. The CFA approach also allowed for the testing and comparison of multiple factor structures to determine the most appropriate structure, such as whether the pornography factors were best treated as related but distinct from one another, or whether they are best modeled as factors nested within a general pornography use factor. The first factor structure tested was a multifactorial first-order oblique structure (see Fig. 2) designed to treat each factor as related but distinct from one another. The second model was a second-order hierarchical model with the first-order factors split off into four higher-order factors based on the themed grouping of factors outlined in Table 1, namely frequency, craving and attractive porn were loaded onto a broad factor reflecting drive to use; secrecy, masturbation, prefer porn and replace partner were loaded onto a broad factor reflecting unhealthy context; joint use, relational content and sex education were loaded onto a broad factor reflecting perceived positives; and aggressive content, nonconsensual content and nonmonogamous content were loaded onto a broad factor reflecting harmful content (see Fig. 3). The third model tested was another second-order hierarchical structure, where the first-order factors were loaded onto a general pornography use factor (see Fig. 4). Finally, a third-order model was tested where the four broad second-order factors from the second model were subsumed into one general pornography use factor (see Fig. 5). To confirm the best-fitting structure from the four models, chi-square difference tests of model fit were conducted (see Table 2).

**Table 2:** Study 1 Chi-square difference tests for different factor structures of the PURRS

Model	*χ*^2^	df	CFI	TLI	RMSEA	SRMR	Comparison	*χ*^2^*Δ*	df *Δ*
Model 1 (first-order oblique)	1489.16	587	.94	.92	.05	.05	Model 1 and Model 2	386.75*	59
Model 2 (4 broad second-order factors)	1875.91	646	.91	.90	.05	.08	Model 2 and Model 3	446.67*	6
Model 3 (1 global second-order factor)	2322.58	652	.88	.87	.06	.09	Model 1 and Model 3	833.42*	65
Model 4 (1 global third-order factor)	Unidentified								

^*χ*2 = Chi-square; *df* degrees of freedom; *CFI* comparative fit index; *TLI* Tucker–Lewis index; *RMSEA* root-mean-square error of approximation; *SRMR* standardized root mean residual^

^*p < 0.001^

### Results and Discussion

Missing data analysis (Little’s MCAR test: *χ*^2^ [2777] = 2868.02, *p* = 0.11) revealed that the data were missing completely at random. As such, missing data was addressed using multiple imputation with all of the available data (demographics and the PURRS items that were completed) included as predictors.

The restricted exploratory factor analysis (EFA) of the 51 items resulted in 13 interpretable factors with eigenvalues ranging between 6.75 and 1.17. The extracted factors yielded a total of 64.65% of the variance explained. The pattern matrix (see in Online Appendix A) was inspected to determine whether the items that loaded onto specific factors made best theoretical sense, whilst also considering the strength of factor loadings and the degree to which items cross-loaded on multiple factors. Furthermore, with a goal of ensuring that the measure was of a length that would minimize burden on participants (i.e., time taken to complete the measure), efforts were made to limit the number of items to a maximum number of four items per factor. This number of items per factor ensured that each factor would be identified as part of subsequent psychometric evaluation of the measure undertaken as part of CFA (e.g., Brown, 2015). Therefore, in reducing the pool of retained items that were part of the CFA modelling, an item was excluded if it: (1) loaded onto a factor that did not correspond with the common theme evidenced across the other items in the factor; (2) demonstrated a loading below 0.35; (c) evidenced moderate-to-large loadings across more than one factor. Guided by these criteria, 13 items were eliminated, leaving 38 items loading onto the 13 factors.

The 38 items and 13 factors were then subjected to confirmatory factor analysis (CFA). Unit variance identification was used, and error terms were not free to covary. Assessment of model fit was determined using guidelines put forward by Hu and Bentler (1999), which suggest that good fit is indicated by a comparative fit index (CFI) and Tucker–Lewis index (TLI) $\ge$ 0.90, root-mean-square error of approximation (RMSEA) $\le$ 0.05, and standardized root mean residual (SRMR) $\le$ 0.06.

As seen in Table 2, the first-order oblique factor structure (Model 1; see Fig. 2) demonstrated good fit, with factor loadings ranging between 0.48 and 0.96. The second-order model with the four broad factors (Model 2; see Fig. 3) demonstrated acceptable fit, with first-order factor loadings ranging between 0.47 and 0.97 and second-order factor loadings ranging between 0.05 and 0.84. The second-order model with one global factor (Model 3; see Fig. 4) demonstrated poor fit, with first-order factor loadings ranging between 0.46 and 0.98 and second-order factor loadings ranging between 0.13 and 0.66. Finally, the third-order factor structure (Model 4) was unidentified and thus could not be estimated.

Chi-square difference tests confirmed that Model 1 was of significantly better fit compared to all the other models (see Table 2). In addition, the 13 factor first-order model demonstrated good internal consistency, with H coefficients ranging between 0.75 and 0.94 (frequency = 0.94; attractive porn = 0.93; craving = 0.81; masturbation = 0.75; prefer porn = 0.85; secrecy = 0.78; replace partner = 0.81; joint use = 0.93; sex education = 0.76; relational content = 0.90; aggressive content = 0.82; nonconsensual content = 0.94) for all but one factor (nonmonogamous content = 0.63).

These results suggest that the 13 first-order factors reflect related but distinct factors (further evidenced by the low-to-moderate correlations found between the majority of factors [see Table 3]) that appear to capture the breadth of dimensions that constitute pornography use in romantic relationships. Model 3 and Model 4 suggest that these factors cannot be reduced to a single broad factor, and this speaks to the importance of developing measures such as the PURRS which take a multidimensional approach to the assessment of pornography use. Our findings do, however, highlight that there may be a case for the presence of higher-order factors based on the themes identified in our initial survey of the literature. These four factors are drive to use, unhealthy context, perceived positives and harmful content, and at the higher-order level, these factors again appear to reflect related but distinct higher-order constructs. However, this higher-order model, while achieving acceptable fit, was not as good a fit as the 13 factor first-order model. Thus, our findings suggest that there are two factor structures that achieve acceptable to good fit that balance measurement precision on the one hand, with increasing parsimony on the other (Table 4), which has implications for researchers. On the one hand, if research is focused on understanding the role of each of the factors as either predictors or outcomes in relationship research, then it is indeed encouraged and preferred that the PURRS be scored and analyzed at the first-order level. However, if researchers have a theoretical interest in the broad factors as predictors or outcomes, or have sample sizes that preclude the running of analytic models that include all 13 facets, then it is justifiable to estimate higher-order subscales and include these in data analytic procedures.

**Table 3:** Correlations between the first-order factors of the PURRS

	1	2	3	4	5	6	7	8	9	10	11	12	13
1. Frequency													
2. Attractive porn	.34												
3. Craving	.44	.23											
4. Masturbation	.27	.21	.29										
5. Prefer Porn	.10	.09	.34	.05									
6. Replace Partner	.16	.07	.27	.11	.46								
7. Secrecy	.12	.08	.26	.14	.38	.50							
8. Relational Content	.01	.02	–	–	–	–	− .14						
9. Sex education	.13	.21	.25	− .04	.18	.06	.05	.15					
10. Joint Use	− .06	.02	.02	− .25	− .08	− .25	− .51	.15	.05				
11. Nonconsensual Content	.08	.04	.21	− .04	.27	.08	.04	− .15	.13	.02			
12. Nonmonogamous Content	.19	.18	.31	.16	.30	.15	.09	− .11	.06	.06	.37		
13. Aggressive Content	.09	.12	.20	.03	.20	− .05	− .07	− .14	.15	.15	.67	.38

**Table 4:** Correlations between the second-order factors of the PURRS

	1	2	3	4
1. Drive to use				
2. Unhealthy context	.50			
3. Perceived positives	− .00	− .64		
4. Harmful content	.31	.11	.10	

### Study 2

Study 2 had two aims. The first aim was to cross-validate the best fitting model identified in Study 1 (i.e., the first-order oblique model). The second aim was to assess the criterion-related validity of the PURRS. To address this second aim, we focused the assessment of criterion-related validity on a series of variables that past research suggests is associated with pornography use. Below, we provide a concise justification regarding the associations expected between the PURRS factors and each of the constructs included as part of Study 2 to assess the criterion-related validity of the PURRS.

First, pornography use has consistently been linked to endorsing unrealistic expectations about sex and sexual partners (Grov et al., 2011; Kohut et al., 2017; Regnerus, 2019; Séguin et al., 2018). Exposure to sexual stimuli depicting extremely attractive people engaging in highly satisfying and intense sexual experiences in which they demonstrate a high degree of sexual prowess and expertise is assumed to be internalized by the pornography user. This internalization is in terms of unrealistic standards and expectations regarding sexual experiences (Séguin et al., 2018; Wright et al., 2019). In turn, pornography users may be more likely to compare their partner to unrealistic standards, and thus, experience sexual encounters as disappointing or dissatisfying when partners do not meet these exceptionally high standards (Wright et al., 2017, 2018). If exposure to such stimuli is more frequent, then it may increase the cognitive accessibility of these unrealistic expectations as well as greater internalization of these standards and expectations (Wright, 2011). On this basis, it is expected that the PURRS factors termed attractive porn and frequency will be positively associated with upward comparisons of one’s partner with pornography.

Second, extensive research has been conducted into pornography use and pornography addiction, with a recent measurement review identifying 13 existing measures of problematic pornography use that were based on an addiction framework (Fernandez & Griffiths, 2021). Though some researchers still dispute the term (e.g., Ley et al., 2014; Prause & Williams, 2020), pornography addiction, like other behavioral addictions such as gambling, consists of several distinct but closely related factors. These include frequency of use, craving, loss of control, concern about one’s use, and impaired functioning (Petry, 2006). Thus, given they are both indicators of addiction, it is expected that the PURRS factors termed frequency and craving will be positively associated with a measure assessing the other aspects of pornography addiction.

Third, a consistent finding in the literature is the negative association between pornography use and the quality of people’s romantic relationships. For example, a large secondary analysis of archival data conducted by Perry (2020b) found that pornography use was frequently associated with poorer relationship quality. Whilst one’s relationship quality might be impacted by a number of pornography use factors, relationship quality is most likely to be impacted by factors that are relationship-oriented by nature, such as preferring to use pornography over having sex with one’s partner, keeping one’s pornography use hidden from one’s partner, or using pornography in place of sex with one’s partner. Masturbation has also been noted as one of the reasons for the negative association between pornography use and relationship quality (Perry, 2020a). This is because masturbation is considered to be a physical substitute for sex with one’s partner, leaving one less motivated to have sex with their partner as they have already experienced sexual release (Ley et al., 2014; Paterson et al., 2014; Perry, 2019). Therefore, it is expected that the PURRS factors termed prefer porn, secrecy, masturbation and replace partner will all be negatively associated with relationship quality.

Fourth, a small but significant positive correlation is consistently found between pornography use and the enactment of negative relationship behaviors such as sexual aggression (Marshall et al., 2017, 2020; Wright et al., 2016), infidelity (Gwinn et al., 2013; Lambert et al., 2012), and negative communication (Maddox et al., 2011; Poulsen et al., 2013; Willoughby & Leonhardt, 2020). These associations are typically explained using sexual script theory (e.g., Wright et al., 2019). Sexual script theory suggests that people internalize the sexual scripts presented in the pornography they are using, which in turn, increases a person’s propensity to act out these scripts in the real world (Wright, 2011). Thus, if a person is using pornography which depicts negative relationship behaviors such as aggression or infidelity, then according to sexual script theory, they are more likely to endorse and engage in these negative behaviors themselves (Hald et al., 2010; Tokunaga et al., 2019; Wright et al., 2016). Thus, it is expected that the PURRS factors termed aggressive content, nonmonogamous content, and nonconsensual content will be positively associated with the extent to which one engages in these negative relationship behaviors.

Lastly, despite pornography use being associated with a variety of negative relationship behaviors and outcomes, there is also mounting evidence that many people who use pornography believe that it is helpful in improving one’s relationship or sex life (Kohut et al., 2017; Watson & Smith, 2012). Some contend that pornography is a good source of sexual education in that it can increase a person’s sexual repertoire (Hesse & Pedersen, 2017; Rissel et al., 2017). In addition, from a sexual script theory perspective, it is assumed that someone, who uses pornography which depicts sex as a means of expressing love and connection, would be more likely to enact these behaviors themselves (Wright, 2011). Lastly, others suggest that using pornography jointly with one’s partner can enhance a couple’s relationship or sex life by encouraging open communication about sex (Kohut et al., 2017). Therefore, it is predicted that the extent to which one believes that their pornography use improves their relationship and sex life is likely to be positively associated with the PURRS factors termed sex education, relational content and joint use.

### Participants

Study 2 consisted of 765 participants (77.2% men, 19.9% women, 2.9% identified as another gender). Participants ranged from 18 to 76 years of age (*M* = 29.58, *SD* = 8.33) and had been in a romantic relationship for an average of just under six and a half years (*M* = 6.39, *SD* = 6.10). Most of the sample reported being heterosexual (79.3%) or bisexual (15.9%). The sample consisted of people who were married (34.9%), engaged (9.8%), cohabiting (24.7%), steadily dating (28.9%) and casually dating (1.6%). The sample was predominantly Caucasian (79.8%), Hispanic (8%), and Asian (7.7%) and living in the USA (49.9%), Australia (13.2%), the UK (8.4%), and Canada (6.5%). The sample was well-educated with approximately 80% having completed further study following high school, and over three quarters of the sample reported that they did not follow any religion.

### Materials and Procedure

Participants were recruited using the same procedure as Study 1. Participants completed an online survey via Qualtrics which took approximately 25 min to complete and included the final 38-item version of the PURRS from Study 1 (see Online Appendix B), along with measures relating to criterion-related variables which are now described.

#### Partner Comparisons

The Partner Comparisons scale was based on past literature which highlighted that using pornography may result in unrealistic expectations and upward comparisons of one’s partner (Kohut et al., 2017; Séguin et al., 2018; Wright et al., 2019). The measure consisted of three items which asked participants about how one’s partner measured up to those depicted in pornography (“I wish my partner looked more like the people in the pornography I use,” “I wish my partner behaved more like the people in the pornography I use,” and “I wish sex with my partner was more like the sex in the pornography I use”). These items were rated on a 7-point Likert scale ranging from 1 (strongly disagree) to 7 (strongly agree). The three items were averaged to create an overall partner comparisons score, with higher scores indicative of greater desire for one’s sexual experiences and partner to be like those in the pornography used. The measure demonstrated very good internal consistency (H coefficient = 0.87).

#### Pornography Addiction

Pornography Addiction was measured using six items which assessed other aspects of pornography addiction aside from frequency and craving (Petry, 2006). This included loss of control (e.g., “I try to reduce the amount of pornography I use but I am unable to”), concern about one’s level of use (e.g., “I feel concerned about the amount of pornography I use”), and impaired functioning because of one’s use (e.g., “I fail to do what is normally expected of me because of my pornography use”). These items were rated on a 7-point scale ranging from 1 (never) to 7 (all of the time). The six items were averaged to create an overall score of pornography addiction, with higher scores indicative of greater addiction. The measure demonstrated very good internal consistency (H coefficient = 0.89).

#### Relationship Quality

Relationship Quality was assessed using the 18-item Perceived Relationship Quality Components (PRQC; Fletcher et al., 2000) inventory. Items were rated on a 7-point scale from 1 (not at all) to 7 (extremely) and asked about aspects of one’s relationship with their partner including satisfaction, commitment, intimacy, trust, passion, and love. Items can be averaged to create six different subscales made up of 3 items each, or all 18 items can be averaged to create an overall score of relationship quality, with higher scores indicative of greater relationship quality. The measure demonstrated excellent internal consistency in the current sample (H coefficient = 0.96).

#### Partner Maltreatment

The Measure of Partner Maltreatment (MPM; Knox & Karantzas, 2022) consisted of 46 items which ask about various forms of partner maltreatment ranging from the more severe (e.g., IPV, infidelity and sexual coercion) to the more subtle (e.g., conflict avoidance and destructive engagement). This measure was created to address the piecemeal approach typically taken by research on this topic by grouping the myriad of negative and destructive behaviors within romantic relationships under the broader concept of partner maltreatment. In doing so, it accounts for a wide spectrum of behaviors which involve maltreating one’s partner. Items were rated on a 7-point scale ranging from 1 (never) to 7 (always). As part of this scale, 12 subscales can be aggregated, or all of the items can be averaged to create an overall score of partner maltreatment, with higher scores indicative of more frequent enactment of negative relationship behaviors. The measure demonstrated excellent internal consistency in the current sample (H coefficient = 0.97).

#### Perceived Gains

The measure of Perceived Gains included three items adapted from the Short Form of the Pornography Consumption Effects Scale (PCES-SF; Miller et al., 2019a, b) which assessed the extent to which participants believed that pornography use had improved their relationship or sex life (e.g., “Using pornography has improved sex with my partner” and “Using pornography has improved my relationship with my partner”). Each item was rated on a 7-point Likert scale ranging from 1 (strongly disagree) to 7 (strongly agree). The three items were averaged to create an overall score of perceived gains, with higher scores indicative of one perceiving greater gains from their pornography use. The measure demonstrated very good internal consistency in the current sample (H coefficient = 0.88).

### Results and Discussion

Results are split into two different sections. The first section reports results pertaining to the cross-validation of the construct validity of the PURRS using CFA, where the best fitting model presented in Study 1 was re-tested with the sample from Study 2. The second section reports results relating to the criterion-related validity of the PURRS, by examining correlations between the proposed factors within the PURRS and various criterion-related variables.

#### Construct Validity

The findings from Study 2 regarding the construct validity of the PURRS replicated those from Study 1. Confirmatory factor analysis demonstrated that the first-order oblique model (Fig. 5) was a good fit of the data (χ^2^ [587] = 1531.94, CFI = 0.94, TLI = 0.92, RMSEA = 0.05, SRMR = 0.05), with factor loadings ranging between 0.50 and 0.95. In addition, the 13 factor first-order solution again demonstrated good internal consistency, with H coefficients ranging between 0.77 and 0.94 (frequency = 0.94; attractive porn = 0.93; craving = 0.81; masturbation = 0.83; prefer porn = 0.86; secrecy = 0.80; replace partner = 0.80; joint use = 0.91; sex education = 0.77; relational content = 0.90; aggressive content = 0.81; nonconsensual content = 0.94) for all but one factor (nonmonogamous content = 0.63). These factor loadings and H coefficients were similar to those found in Study 1.

#### Criterion Validity

Criterion-related validity was evaluated using the first-order 13 factor oblique model (Fig. 5). Specifically, the correlations between the 13 first-order PURRS factors and the criterion-related variables—partner comparisons, pornography addiction, relationship quality, partner maltreatment and perceived gains—were examined.

The zero-order correlations between these criterion-related variables and the 13 first-order factors of the PURRS are presented in Table 5. Findings were predominantly in line with predictions; attractive porn and frequency were positively correlated with Partner Comparisons; frequency and craving were positively correlated with Porn Addiction; prefer porn, secrecy and replace partner were negatively correlated with Relationship Quality; nonconsensual content and were positively correlated with Partner Maltreatment; and finally, sex education, relational content, and joint use were positively correlated with Perceived Gains. However, in contrast to predictions, nonmonogamous content was not significantly associated with Partner Maltreatment, and masturbation was not significantly associated with Relationship Quality.

**Table 5:** Zero-order correlations between criterion-related variables and PURRS’ first-order factors

	1	2	3	4	5	6	7	8	9	10	11	12	13	14	15	16	17	18
1. Relationship Quality																		
2. Partner Maltreatment	− .43**																	
3. Porn Addiction	− .09*	.22**																
4. Partner Comparisons	− .31**	.29**	.30**															
5. Perceived Gains	.22**	− .09*	− .15**	.06														
6. Frequency	− .11*	.07	.27**	.28**	.15**													
7. Attractive Porn	.03	.01	.06	.30**	.18**	.33**												
8. Craving	− .17**	.19**	.33**	.40^**^	.09^*^	.39^**^	.22^**^											
9. Masturbation	− .07	.03	.08^*^	.02	− .10^**^	.18^**^	.15^**^	.18^**^										
10. Prefer Porn	− .40**	.26^**^	.23^**^	.46^**^	− .08^*^	.09^*^	− .01	.30^**^	.00									
11. Replace Partner	− .47**	.35^**^	.24^**^	.46^**^	− .16^**^	.26^**^	.08^*^	.32^**^	.11^**^	.39^**^								
12. Secrecy	− .27**	.22^**^	.47^**^	.29^**^	− .29^**^	.11^**^	.03	.17^**^	.14^**^	.28^**^	.40^**^							
13. Relational Content	.00	− .10^*^	− .12^**^	− .06	.17^**^	.02	− .01	− .03	− .15^**^	− .04	− .01	− .17^**^						
14. Sex Education	.03	.11^**^	.08^*^	.29^**^	.40^**^	.14^**^	.16^**^	.27^**^	− .08^*^	.12^**^	.04	− .01	.15^**^					
15. Joint Use	.16**	− .01	− .19^**^	− .04	.51^**^	.03	.01	.04	− .23^**^	− .04	− .14^**^	− .41^**^	.14^**^	.18^**^				
16. Nonconsensual Content	− .05	.12^**^	.08^*^	.24^**^	.08^*^	.12^**^	− .04	.22^**^	− .02	.21^**^	.13^**^	.07	− .13^**^	.18^**^	.10^**^			
17. Nonmonogamous Content	− .03	.06	.10^**^	.24^**^	− .02	.13^**^	.17^**^	.26^**^	.08^*^	.16^**^	.12^**^	.06	− .11^**^	.17^**^	.03	.30^**^		
18. Aggressive Content	− .05	.14^**^	.08^*^	.26^**^	.17^**^	.15^**^	.07^*^	.22^**^	.03	.13^**^	.05	− .01	− .23^**^	.19^**^	.19^**^	.62^**^	.31^**^	
Mean	5.88	1.66	2.34	2.70	2.97	4.41	5.15	3.11	6.03	1.94	2.33	3.34	3.24	2.61	1.95	1.78	3.16	2.64
Standard deviation	0.97	0.49	1.14	1.51	1.25	1.34	1.09	1.58	1.08	1.15	1.39	1.61	1.27	1.25	1.04	1.20	1.20	1.25

*p < .05. **p < .01

These findings provide evidence for the convergent and divergent validity of the 13 first-order factors of the PURRS. These criterion-related findings are important because typically, research into pornography use encompasses unidimensional self-report assessments, and in most cases, only measure frequency of use. The utilization of unidimensional measures of pornography use is unlikely to provide a complete and comprehensive understanding of the effects of pornography use on romantic relationships. Further still, the utilization of unidimensional measures may result in erroneous conclusions regarding the impact of pornography use on relationship functioning. For instance, if a researcher was interested in examining the association between pornography use and partner maltreatment and utilized a unidimensional measure which focused on frequency (the most common of existing unidimensional measures), then on the basis of our findings, frequency would have no association with maltreatment. The conclusion drawn would be that pornography use is unrelated to the maltreatment of a relationship partner. However, our findings suggest that there are a wide variety of factors related to pornography use, which are indeed positively and significantly related to partner maltreatment. Specifically, craving, prefer porn, replace partner, secrecy, sex education, nonconsensual content and aggressive content are all PURRS factors that are significantly positively associated with Partner Maltreatment. This example highlights that taking a multidimensional approach to pornography assessment can yield very different conclusions regarding the associations between pornography use and romantic relationships.

In summary, the findings of Study 2 provided further support for the construct validity of the PURRS. Furthermore, tests of criterion-related validity provided evidence for both convergent and divergent validity for the first-order factors comprising the PURRS.

## General Discussion

Recent reviews of the pornography literature have highlighted substantial measurement problems in the field (Kohut et al., 2019; Marshall & Miller, 2019; Short et al., 2012). These reviews have stressed the importance of developing valid and reliable multidimensional measures of pornography use that also include a definition of pornography use. The current paper outlines the development and psychometric evaluation of one such measure, the Pornography Use in Romantic Relationships Scale (PURRS).

The development of the PURRS resulted in the identification of a tool consisting of 13 first-order factors reflecting the multidimensional nature of pornography use in romantic relationships (see Online Appendix C for definitions of each factor). As outlined in Table 1, these factors can be clustered into broader themes, and they have consistently been highlighted by recent literature in the field. Study 1 and Study 2 used two independent samples to establish the construct validity of the measure and found that the 13 factor first-order model demonstrated good fit and internal consistency. However, researchers who wish to conduct research on specific aspects of pornography use can choose to include the particular subscales that best address their research questions. Moreover, although some researchers may wish to examine all aspects of pornography use, theoretical and statistical considerations may preclude running analyses involving all 13 factors. In these circumstances, we suggest that researchers can aggregate across the 13 factors to calculate higher-order scale scores in line with the higher-order structure tested and evaluated in Study 1. Although the findings of Study 1 suggested that the first-order solution was the best-fitting model, we also found that the higher-order structure entailing drive to use, unhealthy context, perceived positives, and harmful content was of acceptable fit. Therefore, although we recommend that researchers conduct analyses at the first-order level, when there are issues of statistical power and theoretical considerations that warrant a higher-order conceptualization of pornography use, we endorse the use of higher-order scale scores.

Study 2 extended the psychometric evaluation of the PURRS by assessing the criterion-related validity of the scale. The findings suggested that the PURRS factors were associated with criterion-related variables that were largely in line with predictions, providing evidence for the divergent and convergent validity of the PURRS. In summary, the PURRS factors were found to be positively correlated with upward comparisons of one’s partner with pornography, symptoms of pornography addiction, beliefs that using pornography has improved one’s relationship, and acts of partner maltreatment, whilst being negatively correlated with relationship quality. Below, we provide interpretation of these evidenced associations.

The first-order factors termed attractive porn and frequency were positively associated with upward comparisons of one’s partner with pornography. Both scholars (e.g., Muusses et al., 2015; Wright et al., 2019) and pornography users (e.g., Kohut et al., 2017) have cited this as an outcome of pornography use for some time, but the current study provides the first quantitative evidence of this association. It is widely assumed that pornography depicts extremely attractive people engaging in intense sexual experiences in which they demonstrate sexual prowess and expertise. The findings of Study 2 also provide the first empirical support for this, with most participants scoring at the high end of the attractive porn subscale (mean of 5.20 out of 7; see Table 5). The attractive porn subscale assesses the extent to which the respondent evaluates the people in the pornography they use as attractive and good at having sex. It is possible that these evaluative judgments are then likely to be internalized by the pornography user in terms of unrealistic standards and expectations regarding sexual experiences (Séguin et al., 2018; Wright et al., 2019). These unrealistic standards may be one reason that pornography use is associated with lower sexual satisfaction (Wright et al., 2017, 2018), highlighting the importance of the attractive porn subscale. Additionally, if exposure to such stimuli is more frequent, then it is likely to increase the cognitive accessibility of these unrealistic expectations and lead to greater internalization of these standards and expectations (Wright, 2011). Evidently, frequency has been assessed across decades of pornography research for good reason, and the PURRS frequency factor takes into account one’s level of use over the past year, past month, and past week.

Unsurprisingly, the first-order factors termed frequency and craving were associated with other symptoms of pornography addiction. This included loss of control, concern about one’s use, and impaired functioning. Whilst pornography addiction is not a relationship variable per se, pornography addiction is likely to have a considerable impact on the relationship partner and the romantic relationship more generally (Duffy et al., 2016; Stewart & Szymanski, 2012). Thus, it is important to assess the frequency of one’s use and the extent to which one craves the use of pornography within the context of romantic relationships.

Whilst the preceding two paragraphs underlined the value of the PURRS factors reflective of one’s drive to use (frequency, attractive porn, craving), first-order factors reflective of unhealthy context (replace partner, prefer porn and secrecy) were all associated with poorer relationship quality. These contextual factors are rarely assessed empirically, but the current findings emphasize the need to account for such factors when investigating the impact of pornography use on key relationship outcomes. As a case in point, it is well established that a small but consistent association exists between pornography use and various behaviors which comprise partner maltreatment (Lambert et al., 2012; Marshall et al., 2017, 2020; Poulsen et al., 2013; Wright et al., 2016). The typical explanation for this finding relates to sexual scripting; if pornography depicts people engaging in maltreatment behaviors toward their sexual partner, the person using the pornography internalizes these scripts, and then, the user is more likely to act out these scripts in their own life (Wright, 2011).

Study 2 provided further evidence for this sexual scripting effect, as two of the harmful content factors—nonconsensual content and aggressive content—were weakly but significantly positively associated with acts of partner maltreatment. However, the contextual factors prefer porn, replace partner, secrecy were more strongly associated with greater partner maltreatment than these harmful content factors. This indicates that though sexual scripting may be partly responsible for pornography’s association with partner maltreatment, it is not a sufficient explanation for this finding. Moreover, the contextual factors may help to elucidate why past research has found that using pornography which does not depict maltreatment behaviors is still associated with greater levels of maltreatment behaviors (Wright et al., 2016).

Finally, the first-order PURRS factors that reflect the perceived positives of pornography use (sex education, relational content, joint use) were all positively associated with participants’ reports that their pornography use was good for their sex life and relationship. This suggests that pornography users do indeed perceive these factors to be positive aspects of pornography use. Nevertheless, results from Study 2 indicated that sex education was significantly associated with greater acts of partner maltreatment and increased upward comparisons of one’s partner with pornography. These findings might suggest that though pornography users and several scholars perceive sexual education to be a positive aspect of pornography use, there may be negative consequences in seeking sexual education from pornography. If pornography is perceived to be a source of sexual education, it may set an unrealistic standard of how sex or sexual partners should be, and that most partners will inevitably fall short of (Wright et al., 2019). Moreover, if the pornography being used depicts maltreatment, then perceiving the pornography as sexual education may be more likely to increase endorsement of these behaviors (Peter & Valkenburg, 2006), which, in turn, may increase the likelihood of one engaging in partner maltreatment. Consequently, these findings indicate that the assumption that using pornography for the purposes of sexual education is a good thing for romantic relationships may be partly disputed, or at the very least, over-stated.

### Strengths of the Pornography Use in Romantic Relationships Scale

In addition to demonstrating evidence of internal consistency, construct validity, convergent validity, and divergent validity, the PURRS significantly advances the measurement of pornography use in three main ways.

First, the PURRS presents a definition of pornography use to participants prior to completing the measure and again midway through the measure (see Online Appendix B) to ensure that all participants answer the scale items with the same definition in mind. This definition retains all the important definitional aspects outlined by Kohut et al. (2019; see introduction of this paper), whilst also including the sexually arousing function of pornography, and material depicting sexual activity other than sex such as masturbation. In doing so, this definition embodies lay peoples’ concept of pornography as well as that of leading pornography researchers. However, as the field of pornography research evolves, there may be a need to further advance upon, or revise, the definition we have presented in this paper as part of the PURRS. If this were to occur, we believe that the items comprising the PURRS would continue to be appropriate for administration as they capture many relevant aspects of pornography use. Furthermore, the structural validity of the measure is underpinned by the item-level variance–covariance matrix. Thus, we believe that any changes to the definition is unlikely to render the existing PURRS items as unsuitable for assessing pornography use in romantic relationships.

Second, the PURRS is a multidimensional measure of pornography use. In addition to the most common dimension of pornography use assessed—that of frequency—the PURRS assesses a dozen different aspects of pornography use in romantic relationships which have been repeatedly highlighted by research over the past decade (see Table 1). Therefore, the measure provides researchers with both a more comprehensive and nuanced assessment and understanding of pornography use. In doing so, the PURRS addresses another major measurement problem in the pornography literature: a lack of multidimensional measures of pornography use.

Third, the PURRS is the first measure specifically developed to assess pornography use in romantic relationships. Numerous subscales of the PURRS specifically assess the use of pornography in reference to one’s romantic partner—for example, using pornography to replace a lack of love and intimacy with one’s partner (replace partner), engaging in pornography use that is not disclosed to one’s partner (secrecy), or using pornography with one’s partner (joint use). Given the field’s growing interest in examining and understanding the antecedents and consequences of pornography use in romantic relationships (Willoughby et al., 2020), a measure that assesses the construct within a relational context is important in providing an ecologically valid means of assessing pornography use in romantic relationships.

### Limitations and Future Directions

Although the PURRS represents a significant advancement in the measurement of pornography use, there are some research limitations that need to be acknowledged. A limitation related to Study 2 is that we did not include a stand-alone measure of sexual functioning in our assessments of criterion-related validity. This lack of a criterion-related variable specifically designed to assess sexual functioning may explain why masturbation was not significantly associated with any of the criterion-related variables. Perhaps masturbation to pornography is associated more with sexual variables such as sexual satisfaction or sexual frequency. This is certainly a question to be answered by future research using the PURRS. Nevertheless, it is worth noting that our measure of relationship quality includes an assessment of passion, which is made up of three items (e.g., “How passionate is your relationship?”, “How lustful is your relationship?”, and “How sexually intense is your relationship?”). Given the number of criterion variables tested as part of Study 2, we limited our primary analyses regarding criterion-related validity of relationship evaluations to relationship quality overall. However, supplementary analyses (see Online Appendix D) demonstrate that the passion subscale (compared to all other subscales of relationship quality) demonstrated the largest associations with the PURRS factors (including masturbation). These supplementary analyses at least provide some initial empirical support for the inclusion of the masturbation factor in the PURRS and indicate that several PURRS factors are negatively associated with aspects of sexual satisfaction, findings that are in line with the past research (Wright et al., 2017, 2018).

Another limitation is that both study samples comprised of a low percentage of women. When it comes to pornography use, women have been found to differ considerably from men across a number of variables including frequency, masturbation, and joint use. To account for this, supplementary gender invariance analyses were conducted (see Online Appendix E). Unsurprisingly, there were some item loadings which differed for men and women. However, approximately two-thirds of the item loadings were not significantly different, perhaps suggesting that when a range of factors related to pornography use are taken into account (as opposed to just frequency for instance), men and women are actually more similar than they are different. Regardless, it would be beneficial for future research using the PURRS to recruit a sample with a larger proportion of women.

Similarly, the samples in both studies were relatively homogenous. For instance, participants were predominantly well-educated, of Western culture, and non-religious. Thus, future research would do well to evaluate the psychometric properties of the PURRS using different populations that vary on these and other demographic characteristics.

Lastly, the PURRS is 38-items long, and thus, may be regarded as a somewhat long self-report measure. However, given the multidimensional nature of pornography use, we feel that the number of items that comprise the PURRS strikes a good balance between addressing the many and varied dimensions of the construct while also limiting the number of items addressing each dimension. However, for those researchers who may wish to target specific dimensions of the PURRS as part of their studies, then it is appropriate to merely include the dimensions of interest from the measure rather than administering the whole measure.

### Conclusion

In summary, the current paper demonstrated that the Pornography Use in Romantic Relationships Scale (PURRS) significantly extends on past measures of pornography use by addressing the major measurement problems in the field. The PURRS is a 38-item measure rated on a 7-point scale, which demonstrated good internal consistency, construct validity and criterion validity. It is a multidimensional measure, best modeled by 13 first-order factors which all assess different aspects of pornography use, though a second-order model with four broad factors may also be used. It provides respondents with a definition of pornography use, and it is the first validated measure assessing pornography use specifically in the context of romantic relationships. The PURRS provides a strong basis for the future of self-report measures of pornography use. In particular, use of the PURRS will be advantageous for research that seeks to understand pornography use in romantic relationships.

## Supplementary Information

Below is the link to the electronic supplementary material.

Supplementary file1 (DOCX 34 KB)

Supplementary file2 (DOCX 30 KB)

Supplementary file3 (DOCX 13 KB)

Supplementary file4 (DOCX 21 KB)

Supplementary file5 (DOCX 21 KB)

## Data Availability

The SPSS datasets for the current paper are available at the following link: https://osf.io/m4xyv/?view_only=7b0d1ebc75ac44adbafaff1b9047f2df
