# Supplementary material for: The Development and Validation of the Pornography Use in Romantic Relationships Scale
Source: Arch Sex Behav. 2023 Feb 28;52(4):1799–818. doi: 10.1007/s10508-023-02534-5 (PMC10125950; doi:10.1007/s10508-023-02534-5)
Supplement: Supplementary file 1 — Supplementary file1 (DOCX 34 KB) [file 10508_2023_2534_MOESM1_ESM.docx]

Appendix A. *The pattern matrix from the EFA conducted on the initial 51 items.*

|  | Factor | | | | | | | | | | | | |
| --- | --- | --- | --- | --- | --- | --- | --- | --- | --- | --- | --- | --- | --- |
|  | 1 | 2 | 3 | 4 | 5 | 6 | 7 | 8 | 9 | 10 | 11 | 12 | 13 |
| How often have you used pornography in the past month? | **.95** | .01 | -.04 | .03 | -.01 | -.02 | .00 | .03 | -.02 | .04 | .02 | -.04 | -.03 |
| How often have you used pornography in the past year? | **.89** | -.04 | -.01 | .02 | .01 | -.02 | .03 | .04 | -.01 | -.02 | .00 | .00 | -.01 |
| On how many different occasions have you used pornography in the past week? | **.88** | .00 | -.04 | -.01 | .00 | .01 | .03 | -.05 | -.01 | .02 | -.05 | -.04 | .03 |
| *When you use pornography, how long do you typically use it for?* | .15 | .02 | .08 | .08 | .02 | -.04 | .04 | .06 | .02 | .01 | -.09 | .06 | .05 |
| Someone having something sexual done to them that they don’t want | .01 | **-.93** | -.01 | .02 | .01 | .02 | -.01 | -.01 | -.02 | .02 | -.01 | -.04 | .04 |
| Someone doing something sexual they don’t want to do | .02 | **-.84** | -.04 | .02 | .01 | .05 | -.02 | -.01 | .00 | .09 | .00 | .00 | .11 |
| When I use pornography, I use it with my partner | -.03 | .06 | **.91** | .02 | -.05 | -.01 | -.07 | -.08 | .05 | .01 | .05 | -.05 | .01 |
| I use pornography during sex with my partner | -.02 | .03 | **.82** | .03 | -.01 | .05 | -.05 | .01 | -.04 | .00 | .00 | -.06 | .02 |
| I use pornography because my partner wants me to | .01 | -.09 | **.45** | -.02 | .02 | -.02 | .20 | -.04 | -.01 | .01 | .01 | -.04 | .03 |
| How good-looking are they? | .04 | -.01 | .02 | **.94** | -.02 | .01 | -.07 | -.05 | .01 | .01 | .04 | .05 | -.01 |
| How attractive are they? | .07 | -.03 | .00 | **.89** | .02 | .01 | -.07 | -.02 | .01 | .00 | .01 | .03 | -.02 |
| How sexy are they? | .05 | .00 | .06 | **.86** | .00 | .06 | -.08 | .00 | -.03 | .01 | .00 | .05 | .00 |
| How good are they at having sex? | .00 | -.01 | -.04 | **.48** | -.02 | -.04 | .21 | .03 | -.02 | .02 | .04 | .01 | .11 |
| *How much pleasure do they seem to experience?* | -.06 | .02 | -.04 | .41 | .03 | -.08 | .08 | .04 | .02 | .01 | -.09 | -.18 | -.04 |
| *How sexually aroused do you become whilst using pornography?* | .12 | .01 | .06 | .19 | -.01 | .05 | -.01 | .09 | -.02 | .00 | -.17 | .06 | .10 |
| I use pornography because my partner does not want to have sex | .04 | -.05 | -.04 | .03 | **.90** | .04 | -.05 | .04 | .15 | -.03 | -.01 | -.05 | -.05 |
| I use pornography because it’s easier than trying to have sex with my partner | .07 | .02 | -.02 | .02 | **.67** | .06 | -.07 | .05 | -.24 | -.03 | .03 | .01 | .00 |
| I use pornography because I feel my partner doesn’t love me | -.06 | .03 | .05 | -.01 | **.60** | -.04 | .04 | -.05 | -.02 | .04 | .03 | .07 | .06 |
| *I use pornography because I feel lonely* | -.01 | -.03 | -.04 | -.04 | .45 | -.03 | .10 | .03 | -.07 | -.03 | -.07 | .11 | .01 |
| People having sex as a way of emotionally connecting | -.02 | -.01 | -.05 | -.04 | .03 | **-.93** | .00 | -.01 | .03 | .03 | -.01 | .03 | .07 |
| People having sex as a way of expressing love | .03 | -.10 | -.05 | -.03 | .03 | **-.91** | -.04 | -.03 | -.01 | -.01 | .02 | .00 | -.01 |
| People who are in a relationship having sex | .04 | -.03 | .03 | .00 | .03 | **-.63** | -.04 | .00 | .03 | .02 | .02 | .06 | -.11 |
| People engaging in affectionate behaviour other than explicit sexual activity (for example, hugging or kissing) | -.05 | .09 | -.01 | .01 | -.06 | **-.60** | .01 | -.03 | -.04 | -.05 | .01 | -.09 | .03 |
| *Every person ensuring that those who sexually pleasure them also receive sexual pleasure themselves* | .02 | .12 | .02 | .08 | -.05 | -.46 | .05 | .09 | .04 | -.06 | .03 | -.05 | .04 |
| I use pornography to learn things about sex | .00 | .06 | -.03 | -.01 | .01 | -.02 | **.85** | -.03 | .01 | .02 | -.04 | -.01 | .02 |
| *I use pornography to improve sex with my partner* | .06 | .07 | .25 | -.02 | -.03 | -.01 | .68 | -.05 | .01 | .00 | .00 | .00 | .02 |
| You can learn a lot about sex by using pornography | .05 | -.03 | -.04 | .11 | .03 | .01 | **.64** | .03 | .03 | -.01 | .05 | -.01 | .01 |
| Using pornography teaches me how I should behave when having sex | -.01 | -.08 | -.07 | -.02 | -.04 | .05 | **.57** | .02 | -.03 | .07 | .02 | .02 | .06 |
| *I use pornography out of curiosity* | .00 | .09 | .00 | -.04 | .09 | -.03 | .40 | -.09 | -.06 | -.02 | -.09 | -.02 | .01 |
| *The scenarios in which sex takes place in pornography are not realistic, they are just fantasy* | .00 | .07 | -.05 | -.01 | .02 | .04 | -.16 | -.01 | .01 | .00 | .01 | -.04 | .07 |
| I masturbate whilst using pornography | .00 | .03 | .00 | -.06 | .00 | -.03 | -.04 | **.87** | .06 | .01 | .07 | -.02 | .10 |
| I use pornography to masturbate | .05 | -.02 | .05 | -.01 | .03 | .02 | -.01 | **.70** | .01 | -.02 | -.02 | .01 | -.02 |
| *I focus all of my attention on the pornography whilst I’m using it* | -.03 | .00 | -.10 | .03 | .00 | .00 | -.02 | .50 | -.07 | .05 | -.06 | .03 | -.04 |
| I would prefer to masturbate whilst using pornography than have sex with my partner | -.01 | -.05 | -.03 | -.03 | -.07 | .03 | -.04 | -.01 | **-.83** | .04 | .04 | .04 | .01 |
| Using pornography is more sexually arousing than having sex with my partner | .02 | -.01 | .01 | .02 | .00 | .04 | .01 | -.03 | **-.77** | .05 | -.04 | .10 | .02 |
| I use pornography because it's better than having sex with my partner | .04 | .03 | .00 | .01 | .18 | -.01 | -.02 | .01 | **-.70** | -.04 | .07 | -.02 | .10 |
| *I use pornography to become sexually aroused* | .01 | -.01 | .03 | .03 | .00 | .03 | .12 | .11 | -.26 | -.06 | -.16 | -.09 | -.06 |
| *I plan on using pornography ahead of time* | .03 | -.05 | .14 | -.01 | .02 | -.13 | -.01 | .00 | -.16 | .11 | -.13 | .06 | -.04 |
| Physical aggression (e.g., spanking, slapping, gagging, hair-pulling etc.) | .03 | .13 | -.03 | .03 | -.01 | .00 | .03 | .03 | .01 | **.97** | .01 | -.04 | -.03 |
| Verbal aggression (e.g., name calling, threats etc.) | -.01 | -.17 | .03 | .00 | -.02 | .00 | -.03 | -.02 | -.03 | **.65** | .00 | .00 | .00 |
| Someone being treated as a sex-object/plaything (used for someone else’s sexual pleasure without regard for their feelings) | .02 | -.17 | .01 | .00 | .02 | .15 | .03 | .03 | .00 | **.55** | .00 | .00 | .19 |
| I will use pornography as soon as I get the chance | .10 | -.02 | -.02 | -.04 | .02 | .00 | -.06 | -.05 | -.02 | -.01 | **-.78** | .03 | .09 |
| The thought of using pornography makes me sexually aroused | -.06 | -.04 | -.02 | .03 | .01 | .05 | .03 | .09 | -.02 | .02 | **-.73** | -.01 | -.04 |
| If the situation allowed, I would use pornography right now | .21 | .01 | .01 | -.05 | .03 | .03 | -.01 | .04 | .07 | .01 | **-.66** | .04 | .03 |
| *I feel a rush of excitement when I use pornography* | -.08 | .02 | .00 | .18 | -.01 | -.04 | .07 | .03 | -.08 | .04 | -.43 | .12 | .04 |
| *I use pornography because my partner forces me to* | -.05 | -.12 | .09 | .01 | .08 | -.06 | .09 | -.04 | -.06 | .08 | -.12 | -.06 | .02 |
| I lie to my partner about my pornography use | -.05 | -.01 | -.02 | -.01 | .01 | -.02 | .00 | .01 | -.03 | -.03 | -.08 | **.73** | .05 |
| I actively hide my pornography use from my partner (e.g. lock the door, clear browsing history, use it when they are not around etc.) | -.01 | .05 | -.09 | .01 | .15 | .05 | .04 | .06 | .00 | .01 | -.04 | **.70** | -.06 |
| My partner knows everything there is to know about my pornography use | -.05 | -.01 | .30 | -.06 | -.08 | -.06 | -.03 | .00 | .11 | .09 | -.05 | **-.39** | -.01 |
| People who have just met having sex | -.05 | -.02 | .03 | .01 | .01 | .02 | .01 | .07 | -.04 | .03 | .01 | .00 | **.70** |
| People having an affair (having sex when at least one of them is known to be in a relationship with someone else) | .06 | -.09 | .01 | .00 | .00 | -.04 | .02 | -.03 | -.02 | .01 | -.08 | .01 | **.58** |

| Note: Bolded factor loadings represent those items which were included in the final 38-item version of the PURRS. |
| --- |
