# Supplementary material for: The Development and Validation of the Pornography Use in Romantic Relationships Scale
Source: Arch Sex Behav. 2023 Feb 28;52(4):1799–818. doi: 10.1007/s10508-023-02534-5 (PMC10125950; doi:10.1007/s10508-023-02534-5)
Supplement: Supplementary file 2 — Supplementary file2 (DOCX 30 KB) [file 10508_2023_2534_MOESM2_ESM.docx]

Appendix B. *The final 38-item version of the PURRS.*

The Pornography Use in Romantic Relationships Scale (PURRS)

The following questions ask about pornography use. Your answers will remain both confidential and anonymous, but if you still do not wish to answer a particular question you may skip it without consequence.

USING PORNOGRAPHY means to intentionally look at, watch, read, or listen to sexually arousing material (pictures, videos, films, written text or audio) which depicts nudity and/or explicit sexual behavior.

*This DOES NOT include participating in interactive sexual experiences in person or online, such as a ‘lap-dance’ in a strip club or a live sex chat.*

1. On average, how often have you used pornography in the past year?

| 1 | 2 | 3 | 4 | 5 | 6 | 7 |
| --- | --- | --- | --- | --- | --- | --- |
| Not at all | 1or 2 times a year | 1 or 2 times a month | Weekly | A few times a week | Daily | More than once a day |

*****IF NOT AT ALL IS SELECTED, STOP HERE*****

1. How often have you used pornography in the past month?

| 1 | 2 | 3 | 4 | 5 | 6 | 7 |
| --- | --- | --- | --- | --- | --- | --- |
| Not at all | Once | Fortnightly | Weekly | A few times a week | Daily | More than once a day |

1. On how many different occasions have you used pornography in the past week?

| 1 | 2 | 3 | 4 | 5 | 6 | 7 |
| --- | --- | --- | --- | --- | --- | --- |
| None | 1 | 2 to 3 | 4 to 5 | 6 to 7 | 8 to 10 | More than 10 |

How often are the following statements true for you?

| 1 | 2 | 3 | 4 | 5 | 6 | 7 |
| --- | --- | --- | --- | --- | --- | --- |
| Never |  |  | Sometimes |  |  | All of the time |

1. I masturbate whilst using pornography
2. Using pornography is more sexually arousing than having sex with my partner
3. I use pornography during sex with my partner
4. I lie to my partner about my pornography use
5. I use pornography because it's better than having sex with my partner
6. When I use pornography, I use it with my partner
7. I actively hide my pornography use from my partner (e.g. lock the door, clear browsing history, use it when they are not around etc.)
8. I would prefer to masturbate whilst using pornography than have sex with my partner

The following questions are about the people depicted in the pornography you use. These people may be the people you see in pornographic videos or photos, or the characters that have been described in written or audio pornography.

| 1 | 2 | 3 | 4 | 5 | 6 | 7 |
| --- | --- | --- | --- | --- | --- | --- |
| A little |  |  | Moderately |  |  | Extremely |

1. How sexy are they?
2. How good-looking are they?
3. How good are they at having sex?
4. How attractive are they?

Reminder: USING PORNOGRAPHY means to intentionally look at, watch, read, or listen to sexually arousing material (pictures, videos, films, written text or audio) which depicts nudity and/or explicit sexual behavior.

For each statement, rate to what extent you agree.

| 1 | 2 | 3 | 4 | 5 | 6 | 7 |
| --- | --- | --- | --- | --- | --- | --- |
| Strongly disagree |  |  | Neither agree nor disagree |  |  | Strongly agree |

1. I use pornography to masturbate
2. I use pornography because my partner wants me to
3. My partner knows everything there is to know about my pornography use
4. The thought of using pornography makes me sexually aroused
5. I use pornography because I feel my partner doesn’t love me
6. I use pornography to learn things about sex
7. I will use pornography as soon as I get the chance
8. I use pornography because my partner does not want to have sex
9. You can learn a lot about sex by using pornography
10. If the situation allowed, I would use pornography right now
11. I use pornography because it’s easier than trying to have sex with my partner
12. Using pornography teaches me how I should behave when having sex

How much of the pornography you use clearly depicts the following?

| 1 | 2 | 3 | 4 | 5 | 6 | 7 |
| --- | --- | --- | --- | --- | --- | --- |
| None of the pornography I use |  |  | About half of the pornography I use |  |  | All of the pornography I use |

1. People having sex as a way of emotionally connecting
2. Someone being treated as a sex-object/plaything (used for someone else’s sexual pleasure without regard for their feelings)
3. People having an affair (having sex when at least one of them is known to be in a relationship with someone else)
4. Someone doing something sexual they don’t want to do
5. People engaging in affectionate behaviour other than explicit sexual activity (for example, hugging or kissing)
6. Verbal aggression (e.g., name calling, threats etc.)
7. People who have just met having sex
8. Someone having something sexual done to them that they don’t want
9. People having sex as a way of expressing love
10. Physical aggression (e.g., spanking, slapping, gagging, hair-pulling etc.)
11. People who are in a relationship having sex

**PURRS Scoring Manual**

*All subscales are calculated by summing the scores of the relevant items and dividing by the number of items to compute the mean*

**First Order**

Frequency (3 items): 1, 2, 3

Attractive porn (4 items): 12, 13, 14, 15

Craving (3 items): 19, 22, 25

Masturbation (2 items): 4, 16

Prefer Porn (3 items): 5, 8, 11

Secrecy (3 items): 7, 10, 18*R**

Replace Partner (3 items): 20, 23, 26

Joint Use (3 items): 6, 9, 17

Sex Education (3 items): 21, 24, 27

Relational Content (4 items): 28, 32, 36, 38

Aggressive Content (3 items): 29, 33, 37

Nonmonogamous Content (2 items): 30, 34

Nonconsensual Content (2 items): 31, 35

**Second Order**

Drive to Use (10 items): 1, 2, 3, 12, 13, 14, 15, 19, 22, 25

Unhealthy Context (11 items): 4, 5, 7, 8, 10, 11, 16, 18*R**, 20, 23, 26

Perceived Positives (10 items): 6, 9, 17, 21, 24, 27, 28, 32, 36, 38

Harmful Content (7 items): 29, 30, 31, 33, 34, 35, 37

*Note: *R means reverse coded*
