# Supplementary material for: The Development and Validation of the Pornography Use in Romantic Relationships Scale
Source: Arch Sex Behav. 2023 Feb 28;52(4):1799–818. doi: 10.1007/s10508-023-02534-5 (PMC10125950; doi:10.1007/s10508-023-02534-5)
Supplement: Supplementary file 3 — Supplementary file3 (DOCX 13 KB) [file 10508_2023_2534_MOESM3_ESM.docx]

Appendix C. *Definition of first order PURRS factors*.

| Specific Factor | Definition  *The extent to which…* |
| --- | --- |
| Frequency | …one uses pornography on a regular basis. |
| Craving | …one craves the use of pornography. |
| Attractive porn | …one finds the people in pornography sexually attractive. |
| Secrecy | …one keeps their pornography use secret from their partner. |
| Masturbation | …one’s pornography use is accompanied by masturbation. |
| Prefer porn | …one prefers using pornography to having sex with their partner. |
| Replace partner | …one’s pornography use replaces the love and intimacy provided by one’s partner. |
| Joint use | …one’s pornography use is part of their relationship with their partner. |
| Relational content | …one uses pornography that clearly depicts healthy relationship content. |
| Sex education | …one believes pornography is a source of sexual education. |
| Aggressive content | …one uses pornography that clearly depicts aggressive content. |
| Nonconsensual content | …one uses pornography that clearly depicts non-consensual sexual acts. |
| Nonmonogamous content | …one uses pornography that clearly depicts sex outside of a monogamous context. |
