# Supplementary material for: The Development and Validation of the Pornography Use in Romantic Relationships Scale
Source: Arch Sex Behav. 2023 Feb 28;52(4):1799–818. doi: 10.1007/s10508-023-02534-5 (PMC10125950; doi:10.1007/s10508-023-02534-5)
Supplement: Supplementary file 4 — Supplementary file4 (DOCX 21 KB) [file 10508_2023_2534_MOESM4_ESM.docx]

Appendix D. *Zero-order correlations between PRQC subscales and PURRS’ first order factors.*

|  | 1 | 2 | 3 | 4 | 5 | 6 | 7 | 8 | 9 | 10 | 11 | 12 | 13 | 14 | 15 | 16 | 17 | 18 | 19 |
| --- | --- | --- | --- | --- | --- | --- | --- | --- | --- | --- | --- | --- | --- | --- | --- | --- | --- | --- | --- |
| 1. Satisfaction |  |  |  |  |  |  |  |  |  |  |  |  |  |  |  |  |  |  |  |
| 2. Commitment | .66** |  |  |  |  |  |  |  |  |  |  |  |  |  |  |  |  |  |  |
| 3. Intimacy | .79** | .65** |  |  |  |  |  |  |  |  |  |  |  |  |  |  |  |  |  |
| 4. Trust | .60** | .56** | .56** |  |  |  |  |  |  |  |  |  |  |  |  |  |  |  |  |
| 5. Passion | .60** | .36** | .70** | .29** |  |  |  |  |  |  |  |  |  |  |  |  |  |  |  |
| 6. Love | .72** | .80** | .71** | .58** | .44** |  |  |  |  |  |  |  |  |  |  |  |  |  |  |
| 7. Frequency | -.08 | -.10* | -.12** | -.01 | -.10* | -.08* |  |  |  |  |  |  |  |  |  |  |  |  |  |
| 8. Attractive Porn | .03 | .03 | .04 | .05 | -.01 | .03 | .33** |  |  |  |  |  |  |  |  |  |  |  |  |
| 9. Craving | -.14** | -.12** | -.16** | -.07 | -.19** | -.09* | .39** | .22** |  |  |  |  |  |  |  |  |  |  |  |
| 10. Masturbation | -.02 | -.06 | -.06 | -.01 | -.13** | -.01 | .18** | .15** | .18** |  |  |  |  |  |  |  |  |  |  |
| 11. Prefer Porn | -.36** | -.31** | -.36** | -.16** | -.40** | -.30** | .09* | -.01 | .30** | .00 |  |  |  |  |  |  |  |  |  |
| 12. Replace Partner | -.45** | -.19** | -.46** | -.17** | -.64** | -.22** | .25** | .07* | .32** | .11** | .39** |  |  |  |  |  |  |  |  |
| 13. Secrecy | -.22** | -.12** | -.27** | -.05 | -.38** | -.14** | .11** | .02 | .17** | .14** | .28** | .40** |  |  |  |  |  |  |  |
| 14. Relational Content | -.02 | .01 | .02 | -.03 | .00 | .04 | .02 | -.01 | -.03 | -.15** | -.04 | -.01 | -.17** |  |  |  |  |  |  |
| 15. Sex Education | .00 | -.05 | .05 | .01 | .08* | .02 | .14** | .16** | .27** | -.08* | .11** | .04 | -.01 | .15** |  |  |  |  |  |
| 16. Joint Use | .13** | .09* | .13** | .05 | .20** | .10** | .03 | .01 | .04 | -.23** | -.03 | -.14** | -.41** | .14** | .18** |  |  |  |  |
| 17. Nonconsensual Content | -.04 | -.08* | -.06 | -.02 | -.01 | -.02 | .12** | -.04 | .21** | -.02 | .21** | .13** | .07 | -.13** | .18** | .10** |  |  |  |
| 18. Nonmonogamous Content | -.06 | -.05 | -.05 | .00 | -.01 | -.02 | .13** | .17** | .26** | .08* | .15** | .12** | .06 | -.11** | .17** | .03 | .30** |  |  |
| 19. Aggressive Content | -.05 | -.08* | -.08* | -.07 | .06 | -.07 | .15** | .07* | .21** | .03 | .13** | .05 | -.01 | -.23** | .19** | .19** | .62** | .31** |  |

Note: * *p* < .05. ** *p* < .01.
