# Supplementary material for: The Development and Validation of the Pornography Use in Romantic Relationships Scale
Source: Arch Sex Behav. 2023 Feb 28;52(4):1799–818. doi: 10.1007/s10508-023-02534-5 (PMC10125950; doi:10.1007/s10508-023-02534-5)
Supplement: Supplementary file 5 — Supplementary file5 (DOCX 21 KB) [file 10508_2023_2534_MOESM5_ESM.docx]

Appendix E. *Results of the gender invariance test for the PURRS using CFA standardized factor loadings.*

|  |  |  | Factor loadings | |
| --- | --- | --- | --- | --- |
| Item No. | Item | Factor | Women | Men |
| **1** | **On average, how often have you used pornography in the past year?** | **Frequency** | **.85** | **.91** |
| 2 | How often have you used pornography in the past month? | Frequency | .94 | .93 |
| **3** | **On how many different occasions have you used pornography in the past week?** | **Frequency** | **.89** | **.83** |
| 4 | I masturbate whilst using pornography | Masturbation | .80 | .68 |
| **5** | **Using pornography is more sexually arousing than having sex with my partner** | **Prefer Porn** | **.81** | **.83** |
| 6 | I use pornography during sex with my partner | Joint Use | .83 | .88 |
| 7 | I lie to my partner about my pornography use | Secrecy | .71 | .52 |
| **8** | **I use pornography because it's better than having sex with my partner** | **Prefer Porn** | **.79** | **.78** |
| 9 | When I use pornography, I use it with my partner | Joint Use | .88 | .90 |
| 10 | I actively hide my pornography use from my partner (e.g. lock the door, clear browsing history, use it when they are not around etc.) | Secrecy | .80 | .87 |
| **11** | **I would prefer to masturbate whilst using pornography than have sex with my partner** | **Prefer Porn** | **.78** | **.85** |
| **12** | **How sexy are they?** | **Attractive Porn** | **.81** | **.90** |
| 13 | How good-looking are they? | Attractive Porn | .92 | .92 |
| **14** | **How good are they at having sex?** | **Attractive Porn** | **.49** | **.54** |
| 15 | How attractive are they? | Attractive Porn | .92 | .90 |
| 16 | I use pornography to masturbate | Masturbation | .84 | .77 |
| **17** | **I use pornography because my partner wants me to** | **Joint Use** | **.49** | **.53** |
| **18** | **My partner knows everything there is to know about my pornography use** | **Secrecy** | **.64** | **.65** |
| **19** | **The thought of using pornography makes me sexually aroused** | **Craving** | **.69** | **.74** |
| **20** | **I use pornography because I feel my partner doesn’t love me** | **Replace Partner** | **.54** | **.56** |
| **21** | **I use pornography to learn things about sex** | **Sex Education** | **.76** | **.75** |
| **22** | **I will use pornography as soon as I get the chance** | **Craving** | **.85** | **.77** |
| 23 | I use pornography because my partner does not want to have sex | Replace Partner | .65 | .78 |
| **24** | **You can learn a lot about sex by using pornography** | **Sex Education** | **.71** | **.79** |
| **25** | **If the situation allowed, I would use pornography right now** | **Craving** | **.78** | **.75** |
| **26** | **I use pornography because it’s easier than trying to have sex with my partner** | **Replace Partner** | **.87** | **.83** |
| 27 | Using pornography teaches me how I should behave when having sex | Sex Education | .73 | .67 |
| **28** | **People having sex as a way of emotionally connecting** | **Relational Content** | **.91** | **.91** |
| 29 | Someone being treated as a sex-object/plaything (used for someone else’s sexual pleasure without regard for their feelings) | Aggressive Content | .83 | .76 |
| **30** | **People having an affair (having sex when at least one of them is known to be in a relationship with someone else)** | **Nonmonogamous Content** | **.61** | **.65** |
| 31 | Someone doing something sexual they don’t want to do | Nonconsensual Content | .97 | .94 |
| **32** | **People engaging in affectionate behaviour other than explicit sexual activity (for example, hugging or kissing)** | **Relational Content** | **.63** | **.69** |
| **33** | **Verbal aggression (e.g., name calling, threats etc.)** | **Aggressive Content** | **.76** | **.82** |
| **34** | **People who have just met having sex** | **Nonmonogamous Content** | **.68** | **.74** |
| 35 | Someone having something sexual done to them that they don’t want | Nonconsensual Content | .93 | .91 |
| **36** | **People having sex as a way of expressing love** | **Relational Content** | **.89** | **.88** |
| **37** | **Physical aggression (e.g., spanking, slapping, gagging, hair-pulling etc.)** | **Aggressive Content** | **.71** | **.75** |
| **38** | **People who are in a relationship having sex** | **Relational Content** | **.73** | **.71** |
|  |  |  |  |  |

Note: This test was conducted using all of the 312 women who completed the PURRS over both Study 1 and 2, and 312 randomly selected men (156 from Study 1 and 156 from Study 2). The 24 bolded items (63% of the total items) did not have significantly different factor loadings for men and women.
